# A Cluster-Based Dual-Adaptive Topology Control Approach in Wireless Sensor Networks

**DOI:** 10.3390/s16101576

**Published:** 2016-09-25

**Authors:** Jinsong Gui, Kai Zhou, Naixue Xiong

**Affiliations:** 1 School of Information Science and Engineering, Central South University, South Road of Lushan, Changsha 410083, China; kaizhou@csu.edu.cn; 2 Deptartment of Business and Computer Science, Southwestern Oklahoma State University, 100 Campus Drive, Weatherford, OK 73096, USA

**Keywords:** WSNs, virtual MIMO, transmission mode, clustering range, hierarchical topology control

## Abstract

Multi-Input Multi-Output (MIMO) can improve wireless network performance. Sensors are usually single-antenna devices due to the high hardware complexity and cost, so several sensors are used to form virtual MIMO array, which is a desirable approach to efficiently take advantage of MIMO gains. Also, in large Wireless Sensor Networks (WSNs), clustering can improve the network scalability, which is an effective topology control approach. The existing virtual MIMO-based clustering schemes do not either fully explore the benefits of MIMO or adaptively determine the clustering ranges. Also, clustering mechanism needs to be further improved to enhance the cluster structure life. In this paper, we propose an improved clustering scheme for virtual MIMO-based topology construction (ICV-MIMO), which can determine adaptively not only the inter-cluster transmission modes but also the clustering ranges. Through the rational division of cluster head function and the optimization of cluster head selection criteria and information exchange process, the ICV-MIMO scheme effectively reduces the network energy consumption and improves the lifetime of the cluster structure when compared with the existing typical virtual MIMO-based scheme. Moreover, the message overhead and time complexity are still in the same order of magnitude.

## 1. Introduction

The Multi-Input Multi-Output (MIMO) technique can boost network throughput, conserve energy, and improve network coverage, which has been supported by various international standards such as 3rd Generation Partnership Project Long Term Evolution (3GPP LTE), LTE Advanced (LTE-A), the next or fifth generation (5G) cellular networks and IEEE 802.16e and IEEE 802.16m. However, each wireless device has to be equipped with multiple antennas to exploit the benefits of MIMO. Due to the high hardware complexity and cost, implementation of multiple transmitting antennas sometimes cannot be available, especially in small wireless devices (e.g., sensors). Cooperative MIMO emulates the functionality of multi-antenna systems by grouping wireless devices to operate as virtual multi-antenna nodes [1], which is also called Virtual MIMO (VMIMO). It is desirable for single-antenna devices to take advantage of MIMO gains efficiently, which enables one to make use of all the neighboring terminals and amortize the cost of multiple antennas.

There are four transmission modes in VMIMO systems, that is, Single-Input Single-Output (SISO), Single-Input Multi-Output (SIMO), Multi-Input Single-Output (MISO), and Multi-Input Multi-Output (MIMO) [2,3]. Some typical works take full advantage of these four transmission modes, for example, the Clustering and Cooperative Protocol (CCP) [2] and the Cooperative MIMO (CMIMO) [3]. The MIMO mode of the CMIMO is limited to 2 × 2 mode, while that of the CCP is not limited. The authors in [4] have drawn two conclusions: First, the SIMO mode, MISO mode and MIMO mode are more energy-efficient compared with the SISO mode. Second, in many cases, the 2 × 2 (MIMO) mode is much more energy-efficient than 1 × 1 (SISO) mode, 3 × 3 (MIMO) mode and 4 × 4 (MIMO) mode. If more nodes cooperate in the VMIMO transmission, it will be very difficult to achieve accurate time synchronization among these cooperative nodes and implement the efficient management of these nodes [5]. Therefore, it is a suitable to limit the number of cooperative nodes to two.

In the existing works that fully explore the benefits of VMIMO, there are still some problems. First, a node’s transmission capability is not considered in a cluster head selection criteria, which may lead to the node with low transmission capability to be elected as the cluster head. Second, all the cluster members employ the same transmission power when they communicate with their cluster heads, which should be adjusted adaptively according to the distances from them to their cluster heads. Third, the burden of a master cluster head is too heavy since it is responsible for aggregating data sent by other nodes in its cluster and exchanging these data with its slave cluster head besides executing inter-cluster communications and acting as a beacon cluster head. Fourth, a clustering range needs to be set in advance, where the specified value may be inappropriate since it is impossible to know all the nodes’ transmission capability in advance. In this paper, we address these problems, and propose an improved clustering scheme for Virtual MIMO-based topology construction (ICV-MIMO), which can determine adaptively not only the inter-cluster transmission modes but also the clustering ranges.

Our main contributions are as follows. First, we consider both nodes’ Remaining Battery Lifetimes (RBLs) and nodes’ approximate maximum transmission range to build the new cluster head selection criterions. Second, we propose an algorithm to optimize a transmission power of each cluster member. Third, we add a data fusion cluster head to amortize the cooperation overhead of a master cluster head, which does not involves more clustering overhead than the CMIMO since the added cluster head selection is integrated into the confirmation process of cluster membership. Fourth, our scheme can avoid the problem of unreasonable clustering range setting since each node can determine adaptively its clustering range according to the information from its neighboring nodes.

The rest of the paper is organized as follows: Section 2 gives an overview of related works on VMIMO systems for WSNs. This is followed by the detailed ICV-MIMO scheme in Section 3. Evaluation results are then presented and discussed in Section 4. Finally, the paper is concluded in Section 5.

## 2. Related Work

The authors in [6,7] employed virtual antenna arrays to obtain diversity and coding gains, where several single-antenna nodes cooperate on information transmission (or reception) to achieve energy-efficient communications. Then, several Virtual MIMO (VMIMO) systems that exploit the diversity gain using the Distributed Space-Time Block Code (DSTBC) for WSNs were proposed. For example, the authors in [8] studied a cooperative MIMO scheme for single-hop transmissions; the author in [9] proposed a cooperative MIMO scheme for delay and channel estimation; the author in [10] analyzed the energy efficiency and training overhead of cooperative MIMO; the authors in [11] considered the incorporation of data aggregation into cooperative MIMO; the authors in [12] investigated a VMIMO for multi-hop transmissions. Differing from the above VMIMO schemes, the author in [13] exploited the multiplexing gain of VMIMO to reduce the cooperation overhead and the circuit energy consumption, which was realized by using the Vertical-Bell Laboratories Layered Space-Time (VBLAST) technique; the authors in [14] optimized MIMO’s operation under power and delay constraints by using VBLAST for spatial multiplexing. In addition, some literatures studied the VMIMO configuration for decision fusion. For example, in [15], the array-processing techniques over VMIMO channels were applied to distributed energy detection for decision fusion in WSNs, while a performance analysis of the maximum ratio combining decision fusion rule over MIMO channels was presented in [16]. Also, some other literatures employed a massive MIMO (where antenna arrays are placed at base stations) to improve both spectrum utilization and energy efficiency. For example, the work in [17] studies channel-aware decision fusion over a VMIMO channel, while the literature [18] focused on a massive MIMO-based decentralized estimation within the massive MIMO framework.

In all the above-mentioned works, multi-hop communications and clustering for early data aggregation were not taken into consideration, which limits the scalability of these schemes in large WSNs. That is, the data cannot be aggregated in cluster head since planar network architecture is adopted, which is hardly applied to large WSNs. Some works addressed these problems. For example, the authors in [19] proposed the MIMO-LEACH scheme, which extended the LEACH [20] scheme to build a cluster-based cooperative MIMO scheme for multi-hop communications in WSNs. The Basic-CMIMO [21] and the Clustering and Cooperative Protocol (CCP) are the DSTBC-based CMIMO schemes for clustered WSNs. The number of cooperative nodes in each cluster of the Basic-CMIMO is limited to two, whereas the CCP optimizes the number of cooperative nodes in each cluster and selects cooperative nodes so as to minimize the energy imbalance (i.e., the cooperative nodes are selected in such a way that the variance of residual energy is minimized).

However, the CCP requires solving the problem of optimal cooperative node selection, which leads to a combinatorial problem of high computational complexity. Thus, to maintain a reasonable computational overhead, the CMIMO limited its treatment to two cluster heads per cluster. Moreover, to minimize the energy consumption, the transmission mode and power of the CMIMO can be different for different hops in inter-cluster communications. The Cluster Head Rotation Cooperative MIMO (CHR-CMIMO) scheme [22] introduced cluster-head rotation, where cluster members rotate to be cluster heads by inner-cluster selection. However, this scheme leads to frequent changes of inter-cluster communication distances. The Group Collaboration MIMO (GCMIMO) [23] proposed the concept of Cooperative Group (CG), where each CG composed by two source nodes sends the source data directly through virtual MIMO link without forwarding data to the cluster head. Although it prevents the source data from being forwarded repeatedly, it cannot exploit the benefits of the early data aggregation within a cluster since the source data are directly sent to the sink instead of the cluster head.

The main differences between the MIMO-LEACH and the CMIMO are as follows: First, the MIMO-LEACH invokes the LEACH that does not take into account MIMO operations in the clustering process, whereas the CMIMO has its own clustering protocol that exploits MIMO operations in its selecting criteria. Second, three nodes (one cluster head and two cooperative nodes) are needed to aggregate and forward the data in the MIMO-LEACH, whereas only the two cluster heads are the ones that perform these functions in CMIMO. Finally, the four transmission modes are available in the CMIMO for each link between any two clusters. However, in the MIMO-LEACH, the complete transmission mode (i.e., MIMO) does not exist among the network, since one cluster head in a cluster is responsible for aggregating data and broadcasting it to two other cooperative nodes in the same cluster, and then these cooperative nodes are responsible for forwarding the data to the cluster head of a receiving cluster.

The main difference between our scheme (i.e., ICV-MIMO) and the MIMO-LEACH is that the four transmission modes are available in our scheme for each link between any two clusters. Also, the main difference between the ICV-MIMO and the CMIMO is that the ICV-MIMO adds a data fusion cluster head to amortize the cooperation overhead of a master cluster head. Although the ICV-MIMO has one cluster head per cluster more than the CMIMO, it does not involves more clustering overhead than the CMIMO since this cluster head (i.e., a data fusion cluster head) selection is integrated into the confirmation process of cluster membership.

## 3. The Improved Clustering Scheme

In this section, we first give an overview of the section. Then we elaborate the cluster formation and power adjustment for intra-cluster communications in detail. Next, we describe the determination for inter-cluster transmission modes. Finally we discuss the design issues of the scheme in this paper.

### 3.1. Overview

The term “topology control” has been used with the different meanings in the literatures for wireless sensor networks. Some typical topology control methods (e.g., works in [24,25,26,27,28]) only control a flat network topology, but clustering algorithms (e.g., LEACH [29], HEED [30], and the vehicular clustering scheme in [31]) can control a hierarchical network topology. Therefore, several authors also regard clustering algorithms as topology control techniques. However, according to the informal definition of topology control in [32] (i.e., this definition is to dynamically change the nodes’ transmitting range in order to maintain some property of the communication graph), they cannot be classified as topology control mechanisms since the transmit power of nodes is usually not modified by conventional clustering algorithms. With the evolution of clustering mechanisms, the transmit powers in both inter-cluster and intra-cluster should be adjusted adaptively. In a sense, it is basically consistent with the definition in [32].

**Figure 1:**
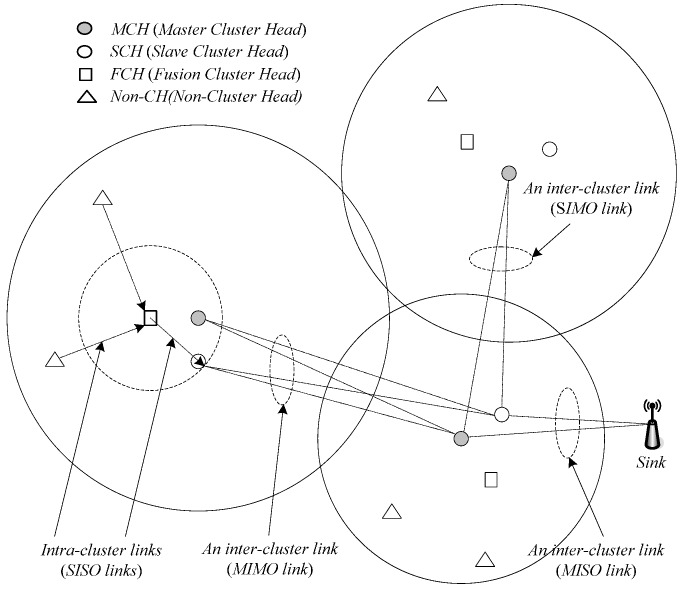
Example for cluster-based network topology.

Our clustering scheme aims at not only minimizing the total energy consumption (transmission plus circuit energies) in inter-cluster communications and improving fair energy consumption in intra-cluster communication but also determine adaptively the inter-cluster transmission modes and the clustering ranges. An example for cluster-based network topology is shown in Figure 1, where each cluster is managed by at most three Cluster Heads (CHs) (i.e., A master CH (MCH), a slave CH (SCH), and a data fusion CH (FCH)), big circles indicate transmission ranges of MCHs, the lines between the nodes are wireless links, and some arrows in links only show the transmission direction of data collection in an intra-cluster.

The MCH and SCH operate as a cooperative multi-antenna node for inter-cluster communications, and the operation consists of three main phases: cluster formation, power adjustment for intra-cluster communications, and determination of inter-cluster transmission modes.

Each cluster sets a FCH to collect data within the cluster. In brief, cluster formation is a distributed process for selecting the CHs of each cluster (the MCH and FCH are mandatory whereas the SCH may or may not be present) and associating non-CH nodes with the corresponding MCH/SCH pairs, where one of the associated non-CH nodes is selected as the FCH. During intra-cluster communications, the FCH is responsible for aggregating data sent by other nodes in its cluster and sending the aggregated data to the corresponding MCH/SCH pairs that may operate as a cooperative multi-antenna node. The inter-cluster communications are carried out by forwarding data from the MCH/SCH pairs of one cluster to those of a neighboring cluster (or directly to the sink/base station), which is built on the basis of inter-cluster topology construction. As mentioned in the CMIMO scheme, an energy-efficient routing algorithm is executed over the topology of virtual nodes to determine an end-to-end inter-cluster path that minimizes the total energy consumption, which is done by running Dijkstra’s algorithm with the weight of a link taken as its total energy consumption. Also, like the CMIMO scheme, each virtual MIMO link, the MCH (or the MCH and SCH) of the receiving cluster selects the optimal transmission mode and transmission power for communication with other CHs, where the mode and power can be different for different hops, depending on the distances between the CHs of neighboring clusters.

### 3.2. Cluster Formation

The cluster formation process consists of the following steps: neighborhood discovery, selecting MCHs, selecting SCHs, determining cluster members, and selecting FCHs.

#### 3.2.1. Neighborhood Discovery

Unlike the CMIMO in which a predefined clustering range is used in neighborhood discovery, each node employs its maximum transmission power to discover its neighbors in our scheme. Moreover, each node will obtain its neighbors’ approximate maximum transmission ranges and their identifiers. To achieve the above goal, the format of the hello message is defined as follows: [*ID_t_*, *e_t_*, *p_t,max_*, *d_t,max_*, *L_t,nei_*={…, (*ID_i_*, *d_i,t_*), …}], where *ID_t_* denotes the identifier of transmitting node *t*; *e_t_*, *p_t,max_*, and *d_t,max_* are the Remaining Battery Lifetime (RBL), maximum transmission power, and approximate maximum transmission range of transmitting node *t* respectively; *L_t,nei_* denotes the list of known neighbors (i.e., the nodes from which the transmitting node *t* has received the hello messages). Each entry in *L_t,nei_* is a tuple with the two elements (i.e., the identifier (e.g., *ID_i_*), and the distance (e.g., *d_i,t_*) from a neighbor (e.g., node *i* ) to itself (e.g., node *t*)).

Once receiving the hello message, a receiving node can measure its receiving power and thus estimate the distance between it and the transmitting node according to the wireless propagation model (e.g., the Equation (2) or Equation (3) in the following text). In our scheme, for simplicity and without the loss of generality, we only consider the two widely used wireless propagation models, namely the free space model and the two-ray ground model [29]. According to the literature [29], the crossover distance is formulated as follows:

(1) $$d_{crossover}=\frac{4\pi \sqrt{L}h_{t}h_{r}}{\lambda }$$

In Equation (1), *h_t_* and *h_r_* are the height of the transmitting and receiving antenna above ground respectively; *λ* is the wavelength of the carrier signal; and *L* is the system loss factor not related to propagation. According to the relation of crossover distance *d_crossover_* and the distance between communicating nodes, one of the two propagation models is chosen. For any link *i*→*j*, the free space model is adopted when *d_i,j_* (i.e., the distance between node *i* and *j*) is less than *d_crossover_*, and thus the wireless propagation formula is as follows:

(2) $$p_{i,j}^{r}=\frac{p_{i,j}^{t}\cdot G_{t}\cdot G_{r}\cdot \lambda^{2}}{{\left(4\pi \right)}^{2}\cdot {\left(d_{i,j}\right)}^{2}\cdot L}$$

If *d_i,j_* is not less than *d_crossover_*, the two-ray ground model is adopted and the corresponding formula is as follows:

(3) $$p_{i,j}^{r}=\frac{p_{i,j}^{t}\cdot G_{t}\cdot G_{r}\cdot {h_{t}}^{2}\cdot {h_{r}}^{2}}{{\left(d_{i,j}\right)}^{4}}$$

In Equations (2) and (3), *p^t^_i,j_* and *p^r^_i,j_* are the transmission power of node *i* and the receiving power of node *j* respectively when node *i* sends packets to node *j*; *G_t_* and *G_r_* are the gains of the transmitting and receiving antenna respectively. Therefore, according to the Equations (2) and (3), the calculating formula for the transmission range from node *i* to *j* is easily derived as follows:

(4) $$d_{i,j}=\{\begin{array}{cc} \sqrt{\frac{p_{i,j}^{t}\cdot G_{t}\cdot G_{r}\cdot \lambda^{2}}{{\left(4\pi \right)}^{2}\cdot p_{i,j}^{r}\cdot L}} & d_{i,j}<d_{crossover}\\ \sqrt[{4}]{\frac{p_{i,j}^{t}\cdot G_{t}\cdot G_{r}\cdot {h_{t}}^{2}\cdot {h_{r}}^{2}}{p_{i,j}^{r}}} & d_{i,j}\geq d_{crossover} \end{array}$$

Our neighborhood discovery process is described in Algorithm 1.

**Algorithm 1.** Neighborhood Discovery (*ND*)
Run at any wireless node (e.g., *u*)
Input: *N_max_*
Output: The identifiers and approximate maximum transmission ranges of all neighbors1.Initialize the variables (e.g., *L_u,nei_* = *Φ*; *d_u,max_* = 0; *N* = 0)2.Use a CSMA/CA scheme to contend for the channel3.**if** succeed in accessing the channel **then**4.  Send [*ID_u_*, *e_u_*, *p_u,max_*, *d_u,max_*, *L_u,nei_*] at the maximum transmission power *p_u,max_*5.  Set the timer *t*_Δ_ as *Δ*6.  Initialize *FLAG*_1_
*and FLAG*_2_ as *false* respectively7. **while** the timer *t*_Δ_ does not expire **do**8. **if** receive [*ID_v_*, *e_v_*, *p_v,max_*, *d_v,max_*, *L_v,nei_*] from any node *v* that is not in *L_u,nei_*
**then**9.   Record the receiving power *p^r^_v,u_* and employ the Formula (4) to compute *d_v,u_*10.   Add (*ID_v_*, *d_v,u_*) to *L_u,nei_*11.     **if**
*d_u,max_* < *d_v,u_*
**then**
*d_u,max_* = *d_v,u_*12.*FLAG*_1_ = *true*13. **else**
**if** receive [*ID_v_*, *e_v_*, *p_v,max_*, *d_v,max_*, *L_v,nei_*] and node *u* is not in *L_v,nei_*
**then**14. *FLAG_2_* = *true*15. **end if**16. **end while**17.  **if** (*FLAG*_2_ == *true*) **then**
*N*++18.  **if** (*N* < *N_max_*) and (*FLAG*_1_ == *true* or *FLAG*_2_ == *true*) **then** go to step 219.**else** go to step 220.**end if**

Take node *u* for an example in Algorithm 1, first, its list of neighbors (i.e., *L_u,nei_*) is initialized as *empty* (see line 1) to prepare for storing the discovered new neighbors. Since *u* does not know any neighbor at the beginning of neighborhood discovery process, its approximate maximum transmission range (i.e., *d_u,max_*) is initialized as *zero* (see line 1). Then *u* uses a CSMA/CA scheme to contend for the channel (see line 2). Once *u* succeeds in accessing the channel (see line 3), it sends the hello message (i.e., [*ID_u_*, *e_u_*, *p_u,max_*, *d_u,max_*, *L_u,nei_*]) at the maximum transmission power *p_u,max_* to discover its 1-hop neighbors (see line 4). After sending the hello message, *u* waits for a fixed duration of time *Δ* to update its list of neighbors (see lines 5~16). During the time Δ, when *u* receives a hello message from a node (i.e., *v*) that is not in *u*’s neighbor list (see line 8), it computes the distance from *v* to *u* (i.e., *d_v,u_*) by means of the Formula (4) (see line 9), and adds this neighbor *v* and the distance *d_v,u_* to the list *L_u,nei_* (see line 10). Also, *d_u,max_* is updated according to the size of *d_v,u_* and *d_u,max_* (see line 11).

The updated neighbor list needs to be sent to all the nodes in the neighborhood of *u*. Also, when *u* receives a hello message from an already known neighbor *v* but *v*’s hello message does not include *u* as a neighbor (see line 13), it needs to resend its hello message, where the number of retransmission for the hello message is kept in the variable *N* that is initialized as *zero* (see line 1) at the beginning of neighborhood discovery process. Therefore, the boolean variables *FLAG*_1_ and *FLAG*_2_ are used to control the transmission of these messages, which are initialized as *false* (see line 6) at the beginning of the time *Δ* and then refreshed as *true* (see lines 12 and 14) if new hello messages are received during the time *Δ*.

After the time *Δ*, if the retransmission for the hello message is needed, the variable *N* is increased by 1 (see line 17). Moreover, if *N* is not less than the predefined value *N_max_*, node *u* terminates the neighborhood discovery process regardless of any value taken by *FLAG*_1_ or *FLAG*_2_ (see line 18).

As discussed in [3], if a network is connected before cluster formation, it is also connected after cluster formation as long as *R_inter_* is not greater than 3*R_intra_*, where *R_inter_* is the inter-cluster transmission range and *R_intra_* is the intra-cluster transmission range. Therefore, if node *i* is elected as a MCH node, it employs the clustering range size that is not more than *d_i,max_*/3, which can make sure that the distance between node *i* and its neighboring MCHs is not more than *d_i,max_*. Due to the difference of the maximum transmission range of each node, we consider the average approximate maximum transmission range in its 1-hop neighborhood (e.g., for node *i*, denoted as *R_i,av_*) to reduce the difference of maximum clustering range that each node can employ.

Through executing Algorithm 1, each node (e.g., *i*) knows not only its approximate maximum transmission range but also those of its neighbors. Therefore, it can estimate the average approximate maximum transmission range in its 1-hop neighborhood by means of the following formula:

(5) $$R_{i,av}=\frac{d_{i,max}+\sum_{j\in L_{i,nei}}d_{j,max}}{|L_{i,nei}|+1}$$

In Equation (5), |*L_i,nei_*| denotes the number of the elements in *L_i,nei_*. By selecting a discount *μ*, a clustering radius for any node (e.g., *i*) is computed by the following formula:

(6) $$R_{i,\mathrm{int}ra}=\mu \cdot R_{i,av}$$

According to Theorem 1 in [3] (i.e., the graph of MCHs generated by the CMIMO protocol is connected if *R_inter_* = 3*R_intra_*), the discount *μ* may be roughly set to 1/3. However, in general, *μ* is a free tunable parameter. For example, in the following simulation setting, when *μ* is set to 0.6, full network connectivity cannot be ensured.

#### 3.2.2. Selecting MCHs

Unlike the CMIMO in which the selection criterion for MCHs is only the nodes’ RBLs, our selection criterion considers both the nodes’ RBLs and the nodes’ approximate maximum transmission range, which is modeled as the weighted sum of RBL and approximate maximum transmission range, since the two metrics are important for selecting cluster heads. The new selection criterion for MCHs is based on the following formula to compute the MCH eligibility metric of each node (e.g., *i*):

(7) $$MM_{i}=w_{e}\cdot \frac{e_{i}}{e_{ref}}+w_{r}\cdot \frac{d_{i,max}}{d_{ref}}$$

In Equation (7), *w_e_* and *w_r_* are the weights of energy and range respectively (they are more than 0 but less than 1, and their sum is equal to 1); *e_ref_* is a reference energy and *d_ref_* is a reference distance, which can be determined and issued by the sink, and thus identical for all nodes. Since the metric unit of RBL is different from that of approximate maximum transmission range, we unify their units by using a ratio form. By an appropriate selection of reference values (i.e., *e_ref_* and *d_ref_* ), *e_i_*/*e_ref_* and *d_i,max_/d_ref_* can be controlled in the same order of magnitude. Therefore, a small change of the weight coefficients (i.e., *w_e_* and *w_r_*) can lead to a more obvious change of the weighted sum, and thus increase the sensitivity of adjustment. If we hope that the elected MCH has more residual energy, we should increase the value of *w_e_* and reduce the value of *w_r_* accordingly, and vice versa. If there is no clear requirement, the two weight variables usually take the roughly same values. Our MCH selection process is described in the Algorithm 2.

**Algorithm 2.** MCH Selection (*MCHS*)
Run at any wireless node (e.g., *u*)
Input: *N_max_*
Output: The identifiers and approximate maximum transmission ranges of all neighbors1.Initialize the state variable *s_u_* as “*undecided*”2.**for** each entry (*ID_v_*, *d_v,u_*) in *L_u,nei_*
**do** initialize the state variable *s_v_* as “*undecided*”3.Compute node *u*’s *R_u,intra_* according to the formulas (5) and (6)4.Compute node *u*’s MCH eligibility metric *MM_u_* according to the Formula (7)5.Initialize *FLAG*_1_ as *true*6.**for** each entry (*ID_v_*, *d_v,u_*) in *L_u,nei_*
**do**7.   **if** (*s_v_* == “*undecided*”) and (*d_v,u_ ≤ R_u,intra_*) **then**8.    Compute node *v*’s MCH eligibility metric *MM_v_* according to the Formula (7)9. **if** (*MM_u_ < MM_v_*) or (*MM_u_ == MM_v_* && *ID_u_* > *ID_v_*) **then**
*FLAG*_1_ = *false*10.   **end if**11.**end for**12.**if** (*FLAG*_1_ == *true*) **then**13.   Determine itself (i.e., node *u*) as the MCH and change the value of *s_u_* as “*decided MCH*”14.   Broadcast the updated *s_u_* to its neighbors to which the distance is not more than *R_u,intra_* from it15.**else**16.   Set the timer *t_τ_* as *τ*17.   Initialize *FLAG*_2_ as *true*18.   **while** the timer *t_τ_* does not expire **do**19.    **if** receive a state variable (e.g., *s_v_*) from any node (e.g., *v*) **then**
20.     **if** the received *s_v_* is “*decided MCH*” **then**21.      Change the value of *s_u_* as “*decided non*-*MCH*”22.      Broadcast the updated *s_u_* to the neighbors to which the distance is not more than *R_u,intra_* from *u*23. *FLAG*_2_ = *false*24. **else** update the initial value of *s_v_* kept in *u* as “*decided non*-*MCH*”25.     **end if**26.    **end if**27.   **end while**28.   **if** (*FLAG*_2_ == *true*) **then** go to step 529.**end if**

After the neighborhood discovery is completed, the selection for MCHs starts. Each node (e.g., *u*) keeps the latest hello messages of all its 1-hop neighbors (obtained during the neighborhood discovery process). All nodes start the clustering process in the *“undecided”* state. Therefore, for each node (e.g., *u*), besides the initialization of its own state variable *s_u_* (see line 1), it also initializes its neighbors’ state variables as the *“undecided”* state (see line 2). Due to the heterogeneity of nodes’ approximate maximum transmission ranges, we employ Equations (5) and (6) to determine the size of clustering radius (see line 3). Also, the selection criterion for MCHs is the node’s MCH eligibility metric, and the node with the highest MCH eligibility metric in its neighborhood can become MCH, so node *u* firstly needs to compute its own MCH eligibility metric (see line 4) and then compares it with those of its 1-hop neighbors (see lines 6~11). The boolean variable *FLAG*_1_ is initialized as *true* (see line 5) for future use. If the MCH eligibility metric of node *u* is not highest, *FLAG*_1_ is changed as *false* (see line 9).

If node *u* has the highest MCH eligibility metric in its neighborhood, it declares itself as the MCH and announces that to its neighbors at the clustering radius *R_u,intra_* (see lines 12~14). Otherwise, it will wait for the *“decided”* messages from its neighbors during the time *τ*, and make the corresponding response (see lines 16~28). At this moment, upon hearing a MCH announcement from any neighbor, *u* switches to a *“decided”* state and announces this new state to its neighbors at the clustering radius *R_u,intra_* (see lines 20~22). Also, the boolean variable *FLAG*_2_ initialized as *true* (see line 17) is changed as *false* (see line 23), which makes *u* stop competing for the role of MCH. However, upon hearing any non-MCH announcement, *u* must keep it (see line 24), which makes the remaining undecided node *u* repeat the above MCH selection process but without considering non-MCH neighbors who are already in the *“decided”* state. After the time *τ*, if *FLAG*_2_ is *true* (see line 28), *u* thinks that it still remain the *“undecided”* state, and thus continues to compete for the MCH.

#### 3.2.3. Selecting SCHs

As mentioned in the CMIMO scheme [3], the purpose of having SCHs is to achieve VMIMO diversity gain during the inter-cluster communications phase by constructing cooperative multi-antenna nodes. However, we propose the improved criteria for SCH selection. Besides the factors considered in the Equation (7), a node’s SCH eligibility metric needs to consider the proximity to the corresponding MCH, which is modeled as follows:

(8) $$SM_{j,i}=MM_{j}\cdot \frac{d_{ref}}{d_{j,i}}$$

In Equation (8), *SM_j,i_* denotes node *j*’s SCH eligibility metric measured by the MCH *i*; *MM_j_* is node *j*’s MCH eligibility metric; *d_j,i_* represents distance between *j* and *i*. The purpose of using the ratio form *d_ref_/d_j,i_* is to make the unit of *SM_j,i_* and that of *MM_j_* consistent. Our SCH selection process is described in Algorithm 3.

After MCHs are selected, the next step is to associate a SCH with each MCH, if possible. Our SCH selection process includes the part run by each MCH node and that executed by each non-MCH node. A MCH node (e.g., *u*) computes SCH eligibility metric for each of its neighbors to facilitate the selection of neighbor with maximum metric value (see lines 1~8), where the purpose of label variable is to prevent the invited node from being invited again when the inviting MCH that is not accepted by the invited node invites other potential SCHs. When knowing the neighbor with the highest SCH eligibility metric value, node *u* sends the “SCH invitation message” to it (see line 9). The Equation (8) is used to compute SCH eligibility metric. This criterion generally favors nodes close to the MCH. At the same time, the RBL value and coverage capacity are also considered. Hence, it is helpful to improve inter-cluster network connectivity needed for coordinating the cooperative MIMO operation. Upon receiving the first invitation message from a MCH, the invited node (e.g., *v*) waits for a fixed duration of time *ζ* before making its decision. If the invited node receives more than one invitation within the time *ζ*, it chooses the closest inviting MCH and sends the “SCH acceptance message” to it (see lines 17~25).

**Algorithm 3.** SCH Selection (*SCHS*)
Run at any MCH node (e.g., *u*)
Input: the hello messages of all neighbors
Output: SCH confirmation message1.**for** each entry (*ID_v_*, *d_v,u_*) in *L_u,nei_*
**do** initialize the label variable *l_v_* as “*uninvited*”2.*SM_max_* = 0; *ID_sch_ = maximum number*3.**for** each entry (*ID_v_*, *d_v,u_*) in *L_u,nei_*
**do**4.  **if** (*d_v,u_ ≤ R_u,intra_*) and (*l_v_* == “*uninvited*”) **then**5.   Compute node *v*’s SCH eligibility metric *SM_v,u_* according to the Formula (8)6.   **if** (*SM_max_ < SM_v,u_*) or (*SM_max_ == SM_v,u_* && *ID_v_* < *ID_sch_*) **then** {*SM_max_ = SM_v,u_*; *ID_sch_ = ID_v_*}7. **end if**8.**end for**9.Send the “SCH invitation message” to the neighbor *ID_sch_*10.**if** eavesdrop the “SCH acceptance message” from the invited node *(*i.e., *ID_sch_*) that does not accept its (i.e., *u*’s) invitation **then**11.  Change the value of the invited node’s label variable as “*invited*”12. Go to step 213.**end if**14.**if** receive the “SCH acceptance message” from the invited node (i.e., *ID_sch_*) that accepts its (i.e., *u*’s) invitation **then**15.  Broadcast the “SCH confirmation message” at its maximum transmission power 16.**end if**
Run at any non-MCH node (e.g., *v*) Input: the hello messages of all neighbors Output: SCH acceptance message17.**if** receive the first “SCH invitation message” from any MCH (e.g., *w*) **then**18.  *d_min_* = *d_v,w_*; *ID_mch_ = ID_w_*19.  Set the timer *t_ζ_* as *ζ*20.  **while** the timer *t_ζ_* does not expire **do**21.   **if** receive the “SCH invitation message” from any MCH (e.g., *u*) **then**22.    **if** (*d_v,u_* < *d_min_*) **then**
**{***d_min_ = d_v,u_*; *ID_mch_ = ID_u_*}23. **end if**24.  **end while**25. Send the “SCH acceptance message” to the neighbor *ID_mch_*26.  Set the timer *t_θ_* as *θ*27.  Initialize *FLAG* as *true*28.  **while** the timer *t_θ_* does not expire **do**29.   **if** receive the “SCH confirmation message” from a MCH that selects *itself* (e.g., *v*) as SCH **then**
*FLAG* = *false*30.  **end while**31.  **if** (*FLAG* == *true*) **then** move to the next step (determining cluster members and selecting FCHs)32.**end if**

If an inviting MCH (e.g., *u*) is not accepted by the invited node (e.g., *v*), it can invite other potential SCHs (see lines 10~13). Upon receiving the “SCH acceptance message”, the MCH whose invitation was accepted confirms this association via the “SCH confirmation message” (see lines 14~16). The purpose of this message is to inform other neighbors of this MCH that they should not expect “SCH invitation messages” from that MCH, so they can move to the next step (determining cluster members and selecting FCHs). The “SCH confirmation message” is sent at the maximum transmission power of this MCH to achieve inter-cluster network connectivity. As mentioned in the CMIMO scheme, the “SCH confirmation message” plays a significant role in MCH-neighborhood discovery, which includes the following fields: MCH ID, SCH ID, and a list of MCHs from which the MCH has already received SCH confirmation messages. The other steps of this discovery approach are similar to those of the neighborhood discovery explained in the Algorithm 1.

A node (e.g., *v*) that is neither an MCH nor an SCH autonomously decides to proceed to the next step (determining cluster members and selecting FCHs) once it has heard “SCH confirmation messages” from all of its MCH neighbors or if no such messages are heard after a duration of time *θ* (see lines 26~31).

#### 3.2.4. Determining Cluster Members and Selecting FCHs

The final step in the cluster formation phase is to have non-CH nodes decide on which cluster to join and then select a cluster member to act as FCH. As mentioned in the CMIMO scheme, since every non-CH node is a neighbor of one MCH (or more MCHs), such a node attempts to associate itself with its closest MCH by sending a “membership request message” that may be replied by the corresponding MCH through sending a “membership list message”.

Unlike the CMIMO scheme, where the MCH assigns the time slots used in the communications between the cluster members and it as well as those between it and the SCH, our Time Division Multiple Access (TDMA) schedule includes the assignment of time slots for the communications between the cluster members and the FCH as well as those between the FCH and MCH/SCH during the intra-cluster communications phase, where the assignment of time slots is also done by the MCH.

In addition, the other difference between our scheme and the CMIMO scheme in this step is that we need to pick a cluster member to act as the FCH. Therefore, the selection criterion for FCHs is an essential prerequisite, which is modeled as follows:

(9) $$FM_{k,j,i}=\frac{e_{k}}{e_{ref}}\cdot \frac{d_{ref}}{\sigma_{k,j,i}}$$

In Equation (9), *FM_k,j,i_* denotes node *k*’s FCH eligibility metric measured by the MCH *i* whose SCH is node *j*; *e_k_* is node *k*’s RBL; *σ_k,j,i_* represents the mean square deviation among *d_k,i_* (i.e., the distance between node *k* and *i*), *d_k,j_* (i.e, the distance between node *k* and *j*), and *d_i,j_* (i.e, the distance between node *i* and *j*). The purpose that *σ_k,j,i_* is considered in FCH eligibility metric is to reduce energy imbalance and energy expended for data transmission between the MCH/SCH and the corresponding FCH. The value of *σ_k,j,i_* is estimated by the following formula:

(10) $$\sigma_{k,j,i}=\sqrt{\frac{{\left(d_{k,i}-d_{k,j,i}\right)}^{2}+{\left(d_{k,j}-d_{k,j,i}\right)}^{2}+{\left(d_{i,j}-d_{k,j,i}\right)}^{2}}{3}}$$

In Equation (10), *d_k,j,i_* denotes the average value of *d_k,i_*, *d_k,j_*, and *d_i,j_*. Our cluster member association and FCH selection process is described in Algorithm 4. In Algorithm 4, a non-CH node first initializes the flag variable of each neighboring MCH as *“unrequested”* (see lines 1~3), and then selects the closest MCH with it from all the neighboring MCHs with *“unrequested”* and attempts to associate itself with this closest MCH by sending the “membership request message” (see lines 4~8). If this non-CH node does not find its *ID* in the “membership list message”, it resends its “membership request message” to the next closest MCH. This process will be repeated until this non-CH node is admitted by a MCH or attempts the maximum number of retransmissions (see lines 9~16). Upon receiving the first “membership request message” from any non-CH node, the MCH computes FCH eligibility metric for this non-CH node according to the Equation (9), adds the *ID* of this non-CH node to the “membership list message”, and sets a fixed duration of time *φ* to allow other non-CH nodes to send their “membership request messages” (see lines 17~21).

**Algorithm**
**4.** Cluster Member Association and FCH Selection (*CMA-FCHS*)
Run at any non-CH node (e.g., *w*) Input: the hello messages of all neighbors Output: the membership request message1.**for** each entry (*ID_u_*, *d_u,w_*) in *L_w,nei_*
**do**2.  **if** the state variable *s_u_* is “*decided MCH*” **then** initialize the flag variable *f_u_* as “*unrequested*”3.**end for**4.*d_min_* = *maximum number*; *ID_mch_ = maximum number*5.**for** each entry (*ID_u_*, *d_u,w_*) in *L_w,nei_*
**do**6.  **if** (*f_u_* == “*unrequested*”) and ((*d_u,w_* < *d_min_*) or (*d_u,w_ == d_min_* && *ID_u_* < *ID_mch_*)) **then**
**{***d_min_ = d_u,w_*; *ID_mch_ = ID_u_*}7.**end for**8.Send the “membership request message” to the neighboring MCH with *ID_mch_*9.**if** receive the “membership list message” from the neighboring MCH with *ID_mch_* (e.g., *y*) **then**10.  **if** find that its *ID* (i.e., *w*’s *ID*) is not in the “membership list message” **then**11.   **if** not more than the given maximum number of resending attempts **then**12.    *f_y_* = “*requested*”13.    Go to step 414.   **end if**15.  **end if**16.**end if**
Run at a MCH node *(*e.g., *u*) which has a SCH node (e.g., *v*) Input: the hello messages of all neighbors Output: the membership list message17.**if** receive the first “membership request message” from any non-CH node (e.g., *w*) **then**18.  Compute node *w*’s FCH eligibility metric *FM_w,v,u_* according to the Formula (9)19.  *FM_max_* = *FM_w,v,u_*; *ID_fch_ = ID_w_*20.  Add *ID_w_* to the membership list message21. Set the timer *t_φ_* as *φ*22.  **while** the timer *t_φ_* does not expire **do**23.   **if** receive the “membership request message” from any non-CH node (e.g., *x*) **then**24.  **if** the number of cluster members is less than the threshold **then**25.     Add *ID_x_* to the membership list message26.     Compute node *x*’s FCH eligibility metric *FM_x,v,u_* according to the Formula (9)27.     **if** (*FM_max_*
*<*
*FM_x,v,u_*) or (*FM_max_ == FM_x,v,u_* && *ID_x_* < *ID_fch_*) **then {***FM_max_ = FM_x,v,u_*; *ID_fch_ = ID_x_*}28.    **end if**29.   **end if**30.  **end while**31.  Declare the node with *ID_fch_* as the FCH through adding it to the membership list message32.  Design a TDMA schedule and add it to the membership list message33.  Broadcast the “membership list message” to its neighbors within the cluster radius34.**else if** a new non-CH wants to join a given MCH and the number of cluster members is less than the threshold **then**35.  Update the TDMA schedule to include this node’s *ID* and add it to the “membership list message”36.  Broadcast the updated “membership list message” to its neighbors within the cluster radius37.**end if**

During the time *φ*, on the one hand, the MCH is willing to accept each non-CH node who sends a “membership request message” to it, unless the number of cluster members exceeds the threshold value that limits the number of non-CH nodes in the cluster; on the other hand, it continues to compute FCH eligibility metric for the cluster members, and records the *ID* of the cluster member with the current maximum FCH eligibility metric (see lines 22~30). After the time *φ*, the MCH declare the cluster member with the maximum FCH eligibility metric as the FCH through adding it to the “membership list message” (see line 31). Also, it designs a TDMA schedule and add it to the membership list message, and then broadcasts the “membership list message” to its neighbors within the cluster radius (see lines 32~33). When a new non-CH node wants to join a given MCH, the TDMA schedule is updated to include this node’s *ID*, which is announced by the MCH via a “membership list message” (see lines 34~36).

### 3.3. Power Adjustment for Intra-Cluster Communications

Unlike the CMIMO scheme in which each cluster member adopts the same transmission power for intra-cluster communication, in our scheme, different cluster member may use different transmission power to communicate with the FCH. Therefore, after cluster formation, power adjustment process has to be executed in our scheme. For any link *i*→*j*, upon knowing node *j*’s receiving sensitivity *p^h^_j_*, node *i* can estimate the minimum transmission power requested to reach node *j* according to the following formula:

(11) $$p_{i,j}^{t}=\{\begin{array}{cc} \frac{p_{j}^{h}\cdot {\left(4\pi \right)}^{2}\cdot {\left(d_{i,j}\right)}^{2}\cdot L}{G_{t}\cdot G_{r}\cdot \lambda^{2}} & d_{i,j}<d_{crossover}\\ \frac{p_{j}^{h}\cdot {\left(d_{i,j}\right)}^{4}}{G_{t}\cdot G_{r}\cdot {h_{t}}^{2}\cdot {h_{r}}^{2}} & d_{i,j}\geq d_{crossover} \end{array}$$

In intra-cluster communications, power adjustment involves the interaction and collaboration between MCH/SCH, FCH, and cluster members, which is described in Algorithms 5 and 6.

Firstly, the MCH sends a power adjusting request (PAREQ) packet at its maximum transmission power during the TDMA slot assigned to the communication between FCH and MCH/SCH (see line 2). This PAREQ packet carries the receiving sensitivity of the MCH, so the node receiving this PAREQ packet can estimate the minimum transmission power requested to reach the MCH according to the Formula (11). In order to receive a power adjusting reply (PAREP) packet from the FCH, the MCH has to wait for the time *σ* (see lines 3~6). After the time *σ*, if such a PAREP packet is not received (see line 7), the MCH will repeat the operations in lines 2~6 (up to a given maximum number of retransmissions). Upon hearing the PAREQ packet from the MCH, the SCH also sends its own PAREQ packet (see lines 8~15), otherwise it will go back (see line 17) to continue to monitor the arrival of the PAREQ packet from the MCH.

The FCH’s operations include the two parts. (a) It adjusts its transmission power used to communicate with the MCH/SCH through receiving the PAREQ packets from the MCH/SCH to obtain their receiving sensitivity values and then using the Equation (11) to estimate the transmission power requested to reach them (see lines 19~39 in Algorithm 5). (b) It reports its own receiving sensitivity to the cluster members through broadcasting a PAREQ packet, and confirms the establishment of links between the SCH and the cluster members through using a power acknowledge list message to reply the PAREP packets from the cluster members (see lines 1~10 in Algorithm 6).

In Algorithm 5, if the transmission power from the FCH to the MCH is different from that from the FCH to the SCH, the largest one is adopted to broadcast a PAREP packet so that the MCH and the SCH all receive it (see lines 23~30). There may not exist a SCH in a cluster. At this time, the goal of power adjustment is to ensure that the FCH can be just connected to the MCH (see line 32). Since the transmission power computed by the Equation (11) is an approximate value, it does not ensure that the sending node can be connected to the corresponding receiving node. Therefore, if the receiving node does not receive the PAREP packet in time, it will retransmit the PAREQ packet. In turn, this makes the sending node retransmit the PAREP packet at the transmission power increased by *ε* (see lines 34~39).

**Algorithm 5.** Power Adjustment for FCH (*PA-FCH*)
Run at a MCH node (e.g., *u*) Input: *N_max_* Output: *NULL*1.Initialize *N* as zero2.Send a PAREQ packet at its maximum transmission power to the FCH (e.g., *w*)3.Set the timer *t_σ_* as *σ*4.**while** the timer *t_σ_* does not expire **do**5.  **if** receive the PAREP packet from the FCH (e.g., *w*) **then** return6.**end while**7.**if**
*N*
**<**
*N_max_*
**then** {*N*++; go to step 2} **else** return **end if**
Run at a SCH node (e.g., *v*) Input: *N_max_* Output: *NULL*8.**if** hear the PAREQ packet from the MCH (e.g., *u*) **then**9.  Initialize *N* as zero10.  Send a PAREQ packet at its maximum transmission power to the FCH (e.g., *w*)11.  Set the timer *t_ρ_* as *ρ*12.  **while** the timer *t_ρ_* does not expire **do**13.   **if** receive the PAREP packet from the FCH (e.g., *w*) **then** return14. **end while**15. **if**
*N*
**<**
*N_max_*
**then** {*N*++; go to step 10} **else** return **end if**16.**else**17.  Go to step 818.**end if**
Run at a FCH node (e.g., *w*) Input: the hello messages of all neighbors and the very small real number *ε* Output: the adjusted transmission power from FCH to MCH/SCH19.**if** receive the PAREQ packet from the MCH (e.g., *u*) **then**20.  Adjust the transmission power *p^t^_w,u_* according to the Formula (11) and initialize *FLAG* as *true*21. Set the timer *t_ψ_* as *ψ*22.  **while** the timer *t_ψ_* does not expire **do**23.   **if** receive the PAREQ packet from the SCH (e.g., *v*) **then**24.    Adjust the transmission power *p^t^_w,v_* according to the Formula (11)25.    **if**
*p^t^_w,u_* > *p^t^_w,v_*
**then**26.    Broadcast a PAREP packet at *p^t^_w,u_* and set *FLAG* as *false*27.    **else**28.     Broadcast a PAREP packet at *p^t^_w,v_* and set *FLAG* as *false*29. **end if**30.   **end if**31. **end while**32. **if** (*FLAG* == *true*) **then** Broadcast a PAREP packet at *p^t^_w,u_*33.**end if**34.**if** receive the repeated PAREQ packet from the MCH (e.g., *u*) or the SCH (e.g., *v*) **then**35.    **if** (*FLAG* == *true*) **then** {*p^t^_w,u_* = *p^t^_w,u_* + *ε*; Broadcast a PAREP packet at *p^t^_w,u_*}36.    **else if**
*p^t^_w,u_* > *p^t^_w,v_*
**then** {*p^t^_w,u_* = *p^t^_w,u_* + *ε*; Broadcast a PAREP packet at *p^t^_w,u_*}37.    **else** {*p^t^_w,v_* = *p^t^_w,v_* + *ε*; Broadcast a PAREP packet at *p^t^_w,v_*} **end if**38.    **end if**39.**end if**

In the Algorithm 6, in order to reduce message overhead, when the FCH receives the first PAREP packet from any cluster member, it must wait for the time *φ* to carry as many cluster members’ *ID*s as possible in the same power acknowledge list message (see lines 4~7). For a cluster member (e.g., *x*), upon receiving the PAREQ packet from the FCH (e.g., *w*), it first adjusts its transmission power (e.g., *p^t^_x,w_*) requested to reach this FCH according to the Equation (11), and then sends a PAREP packet back at the transmission power *p^t^_x,w_* (see lines 11~14). On the other hand, if the cluster member *x* finds that its own *ID* is not in the power acknowledge list message received by it, it retransmits the PAREP packet at the transmission power increased by *ε* since the PAREP packet is not successfully transmitted at the original transmission power (see lines 15~20).

**Algorithm 6.** Power Adjustment for Cluster Members (*PA-CM*)
Run at a FCH node (e.g., *w*) Input: the hello messages of all neighbors Output: *NULL*1.Broadcast a PAREQ packet at its maximum transmission power to the cluster members2.**if** receive the first PAREP packet from any cluster member (e.g., *x*) **then**3.  Add *ID_x_* to the power acknowledge list message4.  Set the timer *t_φ_* as *φ*5.  **while** the timer *t* does not expire **do**6.   **if** receive the PAREP packet from any cluster member (e.g., *y*) **then** add *ID_y_* to the power acknowledge list message7.  **end while**8.**end if**9.Broadcast the power acknowledge list message at its maximum transmission power10.**if** the PAREP packet of any cluster member (e.g., *x*) arrives after the time *φ*
**then** go to step 3
Run at any cluster member (e.g., *x*) Input: the hello messages of all neighbors and the very small real number *ε* Output: the adjusted transmission power from cluster member to FCH11.**if** receive the PAREQ packet from the FCH (e.g., *w*) **then**12.  Adjust the transmission power *p^t^_x,w_* according to the Formula (11)13.  Send a PAREP packet at *p^t^_x,w_* to the FCH14.**end if**15.**if** receive the “power acknowledge list message” from the FCH (e.g., *w*) **then**16.  **if** the *ID_x_* is not in the “power acknowledge list message” **then**17.   *p^t^_x,w_* = *p^t^_x,w_* + ε18.   Send a PAREP packet at *p^t^_x,w_* to the FCH19.  **else** return **end if**20.**end if**


### 3.4. Determination for Inter-Cluster Transmission Modes

We adopt the same method as that in [3] to determine inter-cluster transmission modes, where the purpose is to decide on an appropriate transmission power and antenna mode between a cluster and each of its adjacent clusters. This process is briefly described as follows.

Each MCH is already aware of its neighboring MCHs following the overhearing of the “SCH confirmation messages” in the cluster formation phase. If the MCH of a given cluster wishes to establish VMIMO links with adjacent clusters, it broadcasts a channel probing request (CPREQ) packet. If the given cluster has a SCH, this SCH will send its own CPREQ after it hears this CPREQ.

Upon receiving the two CPREQ packets, the receiving MCHs and SCHs estimate the Channel State Information (CSI) between the source MCH/SCH and the receiving MCH/SCH, and communicate such information to each other. Then the receiving MCH/SCH calculates the minimum power needed to communicate between the MCH/SCHs of the transmitting and receiving clusters using one of four possible modes (i.e., SISO, MISO, SIMO, and MIMO).

The MCH and SCH in each neighboring cluster determine the optimal transmission mode that minimizes the total energy (which includes both transmission and circuit components) among the four modes. Each receiving MCH then sends this information back to the source MCH/SCH (and also to the receiving SCH) via a channel probing response (CPRES) packet.

A network topology consisting of virtual inter-cluster links is constructed, where each link in this topology represents the transmission mode that requires the least amount of energy. Information about the selected transmission power and mode for each link is flooded throughout the network, so that each cluster will eventually have a complete knowledge of all links’ weights, which will be used as input to the path selection algorithm. A minimum-energy routing protocol based on Dijkstra’s algorithm is executed by the source MCH to obtain the path with the minimum total energy among all possible paths between the source MCH/SCH and the sink.

### 3.5. Design Issues of the ICV-MIMO Scheme

We first discuss the connectivity of cluster-based graph consisting of MCHs, SCHs, FCHs, and cluster members:

**Theorem** **1.** For any link i→j in a connected network formed by the ICV-MIMO scheme, where the MCHs of node i and j are node u and v respectively, if there exists the inter-cluster link u→v in the network, the clustering ranges of node u and v are at most d_u,max_/3.

**Proof.**  If there exists the inter-cluster link *u*→*v* in the network, the distance between *u* and *v* (i.e., *d_u,v_*) is not more than *d_u,max_*. According to Lemma 1 in [3], the MCHs of any two neighboring nodes in a connected network are either identical or are within range of 3 times the size of clustering range. Since node *j* is the neighbor of node *i* and node *v* (i.e., the MCH of node *j*) is the neighboring MCH of node *u* (i.e., the MCH of node *i*), the clustering ranges of node *u* and *v* are not more than *d_u,max_*/3. Hence, the theorem is proven. □

The following theorem claims the intra-cluster connectivity of the graph consisting of MCHs, SCHs, FCHs, and cluster members.

**Theorem** **2.** In any cluster with the MCH u, the SCH v, and the FCH w, if any cluster member’s maximum transmission range is not less than 2μ·R_u,av_, the probability that it can be connected to the FCH w is 1. Also, if the FCH w’s maximum transmission range is not less than 2μ·R_u,av_, the probability that it can be connected to the SCH v is 1.

**Proof.**  The MCH *u* is located in the center of the cluster with the radius *μ*·*R_u,av_* according to the Equation (6), where the SCH *v*, the FCH *w*, and cluster members are randomly distributed. Therefore, the distance between any cluster member and the FCH *w* is at most 2*μ*·*R_u,av_*. Also, the distance between the FCH *w* and the SCH *v* is at most 2*μ*·*R_u,av_*. Therefore, the theorem is true. □

Second, we discuss the message and time complexity of the ICV-MIMO scheme in comparison with the CMIMO scheme.

**Theorem** **3.** The message complexity of the ICV-MIMO scheme executed by any node is O(N_nei_), where N_nei_ is the number of this node’s neighbors with which it directly communicates at its maximum transmission power.

**Proof.**  For any given node *u*, no matter what role (e.g., MCH, SCH, FCH, and cluster member) it plays, the maximum message overhead of its executing the ICV-MIMO scheme occurs in the neighborhood discovery phase (i.e., the Algorithm 1). The complexity for any node *u* to broadcast the *hello* messages (see line 4 in the Algorithm 1) is *O*(1) and to receive the *hello* messages (see lines 8~15 in the Algorithm 1) from its neighbors is *O*(*N_nei_*) respectively. This is because the number of transmitting messages is at a constant level while the received messages may come from the neighbors with which *u* directly communicates at its maximum transmission power. So the message complexity of the ICV-MIMO scheme executed by any node is *O*(*N_nei_*). □

**Theorem** **4.** The time complexity of the ICV-MIMO scheme executed by any node is max{O(T_t_), O(N_nei_·T_r_)}, where T_t_ denotes the time duration for the node to successfully access the channel and send a message with a fixed size, and T_r_ is the time duration for the node to successfully receive and process a message with a fixed size.

**Proof.**  For any given node *u*, since the complexity for it to broadcast a message is *O*(1) (see proof of Theorem 3), its time complexity of broadcasting a message is *O*(*T_t_*). Also, since the complexity for node *u* to receive messages is *O*(*N_nei_*) according to Theorem 3, its time complexity of receiving messages from its neighbors is *O*(*N_nei_·T_r_*). Therefore, the time complexity of the ICV-MIMO scheme executed by any node is *max*{*O*(*T_t_*), *O*(*N_nei_·T_r_*)}. □

When compared with the cluster member association in [3], the Algorithm 4 in this paper increases a FCH selection. However, this election process is integrated into the membership determining process, so it does not increase the running cost. Differing from the CMIMO scheme in [3], in this paper, a power adjustment process has to be executed after a cluster formation. Therefore, we increase the Algorithms 5 and 6.

Table 1 presents a comparison of the communication and computational complexities of the ICV-MIMO scheme and the CMIMO scheme. Since *N_deg_* is the number of neighbors at the node’s clustering range, it is less than *N_nei_*. Therefore, the CMIMO scheme slightly outperforms the ICV-MIMO scheme in terms of both the message overhead and the time complexity. In spite of this, their complexity is still in the same order of magnitude.

Then, we discuss the synchronization of the ICV-MIMO scheme. The MCH of the source cluster accesses the medium using CSMA/CA and sends an RTS packet. Upon receiving the RTS, the MCH of the receiving cluster responds with a CTS packet that serves as a synchronization signal for the MCH and SCH in the source cluster, so that they transmit their data simultaneously to achieve MIMO diversity gain.

In addition, a FCH also acts as a beacon cluster head to send a synchronization signal, so that its corresponding MCH and SCH start sending the data simultaneously to the CHs of a receiving cluster. Next, we discuss the reclustering issue of the ICV-MIMO scheme. In most cases, a FCH may be the one that deplete its battery first (before MCHs, SCHs and non-CH nodes) because of its great responsibility in aggregating data, sending it to its MCH/SCH. Therefore, the reclustering request to be sent by any FCH is a viable option.

The key idea for reclustering is that when the RBL of any FCH falls below a specific threshold (e.g., 10% of its initial value), this FCH sends a reclustering message to its MCH, and this MCH forwards it to its neighboring MCHs at its maximum power level. This message will be heard by the requesting MCH’s non-CH nodes, its SCH and FCH, and its neighboring MCHs.

Reclustering messages are similar to the hello messages used in the neighborhood discovery process in the cluster-formation phase. Neighboring MCHs that hear a reclustering message relinquish their cluster head role and invoke a neighborhood discovery process. In fact, if the MCHs do not die, the cluster structure is not destroyed. Even if any FCH dies, the corresponding MCH can take over its work. This can effectively extend the heavy reclustering intervals and thus reduce the cost of reclustering.

Finally, it is worth noting that, the ICV-MIMO scheme only considers large scale fading (i.e., path loss), but ignores small scale fading (e.g., Rayleigh fading). The main reasons are as follows. First, none of very accurate values of maximum transmission ranges is required in our scheme because these values are only regarded as a factor for cluster head election. Therefore, it is enough for us to obtain these values from the same radio propagation formula. And if we take into account small scale fading, our scheme will be more complex and cumbersome.

Second, an exact transmission range is hardly obtained at the cost of scheme complexity in the power adjustment process of intra-cluster communications when we consider both large and small scale fading. For a simplified scheme, we only consider a path loss model in our scheme. However, by increasing or decreasing a small real number *ε*, a rough power obtained from the path loss model turns to an actual required power, as described in Section 3.3. In addition, in the process of power adjustment, reducing transmission power might increase channel error, which results in the increase of retransmission and power consumption. A threshold of retransmission count may be employed to control the power decreasing process.

When the number of retransmissions reaches this threshold (which can be preliminarily determined by the actual measurements), the transmission power is no longer reduced. In addition, in order to prevent the frequent adjustment of transmission power due to the channel quality fluctuation, we may increase a little margin to the resulting transmission power obtained by Algorithms 5 and 6.

## 4. Performance Evaluation

In this section, we evaluate the performance of the ICV-MIMO scheme via simulations. We also compare it with the CMIMO, which resembles the ICV-MIMO scheme in the inter-cluster communication modes.

### 4.1. Energy Model

The energy consumption of inter-cluster communications can be measured by the following formula [3]:

(12) $$E_{x,y}^{bt}=\frac{C_{1}\cdot \gamma (M_{t},M_{r})\cdot {\left(d_{x,y}\right)}^{\alpha_{x,y}}+C_{2}\cdot M_{t}+C_{3}\cdot M_{r}+C_{4}}{R_{b}}$$

In Equation (12), $E_{x,y}^{bt}$ is the total energy consumption per bit in an inter-cluster communication between the cluster *x*’s MCH/SCH and the cluster *y*’s MCH/SCH; *R_b_* is the bit rate; *γ*(*M_t_*,*M_r_*) is the Signal to Noise Ratio (SNR) threshold at the receiver when *M_t_* and *M_r_* antennas are used for transmission and reception respectively; *d_x_*_,*y*_ is the transmitter-receiver distance, which is the maximum value of the four distances between the source MCH/SCH and the destination MCH/SCH in a VMIMO operation; *α_x_*_,*y*_ is path loss exponent (for simplicity, *α_x_*_,*y*_ = 2 when *d_x_*_,*y*_ is less than *d_crossover_*; otherwise, *α_x_*_,*y*_ = 4); *C*_1_, *C*_2_, *C*_3_, and *C*_4_ are circuit-specific constants shown as follows:

(13) $$\{\begin{array}{l} C_{1}=(1+\delta )\cdot N_{o}\cdot B\cdot N_{f}\cdot G_{o}\cdot M_{l}\\ C_{2}=P_{DAC}+P_{mix}+P_{filt}\\ C_{3}=P_{LNA}+P_{mix}+P_{IFA}+P_{filr}+P_{ADC}\\ C_{4}=2\cdot P_{syn} \end{array}$$

In Equation (13), *δ* is a factor that depends on the drain efficiency of the power amplifier and the underlying modulation scheme; *N_o_* is the single-sided thermal noise Power Spectral Density (PSD); *B* is the passband bandwidth; *N_f_* is the receiver noise figure (*N_f_* is defined as *N_r_*/*N_o_*, where *N_r_* is the PSD of the total effective noise at the receiver input); *G_o_* is a constant that depends on the transmitter and receiver antenna gains; *M_l_* is a link margin that compensates for hardware variations and other sources of interference; *P_DAC_*, *P_mix_*, *P_LNA_*, *P_IFA_*, *P_filt_*, *P_filr_*, *P_ADC_*, and *P_syn_* are the power consumption values for the digital-to-analog converter, the mixer, the low noise amplifier, the intermediate frequency amplifier, the active filters at the transmitter and the receiver sides, the analog-to-digital converter, and the frequency synthesizer, respectively.

For a given inter-cluster distance between a pair of neighboring MCHs/SCHs (e.g., *d_x_*_,*y*_), one of the four antenna modes (i.e., SISO, SIMO, MISO, MIMO) is employed based on the principle of minimizing the value of $E_{x,y}^{bt}$. For relatively small distances, a circuit power is more dominant than a transmission power, a single-antenna mode (e.g., SISO) is more energy-efficient than a multi-antenna mode (e.g., one of SIMO, MISO, MIMO). As the transmitter-receiver distance increases, the tradeoff shifts in favor of multi-antenna modes. The energy consumption per bit in the source MCH/SCH (e.g., that of the cluster *x*’s MCH/SCH) is computed by the following formula:

(14) $$\Delta e_{x,y}^{s}=\frac{C_{1}\cdot \gamma (M_{t},M_{r})\cdot {\left(d_{x,y}\right)}^{\alpha_{x,y}}+C_{2}\cdot M_{t}+0.5\cdot C_{4}}{R_{b}}$$

The energy consumption per bit in the destination MCH/SCH (e.g., that of the cluster *y*’s MCH/SCH) is computed by the following formula:

(15) $$\Delta e_{x,y}^{d}=\frac{C_{3}\cdot M_{r}+0.5\cdot C_{4}}{R_{b}}$$

In intra-cluster communications, we also adopt Equations (14) and (15) to compute the energy consumption for sending node and receiving node respectively, where a single-antenna mode is employed, and *d_x_*_,*y*_ is replaced for the distance between sending node and receiving node.

### 4.2. Simulation Metrics and Deployment Settings

Our metrics are as follows: (a) the average transmission power for the cluster members in all the clusters of the network. The metric is computed by $p_{av}=\frac{1}{\left|N\right|}\sum_{i\in N}p_{i}^{t}$, where *N* and |*N*| are the *set* of the cluster members in all the clusters and the number of these cluster members respectively, and *p^t^_i_* is the transmission power from any cluster member *i* to its FCH. (b) The average inter-cluster path hop-count for all the paths from the MCHs/SCHs to the sink. The metric is obtained by $h_{av}=\frac{1}{\left|M\right|}\sum_{i\in M}h_{x}$, where *M* and |*M*| are the cluster *set* and the number of clusters in the network respectively, and *h_x_* is the inter-cluster path hop-count for the path from the MCH/SCH (in the cluster *x*) to the sink. In the ICV-MIMO scheme, each cluster member first transmits its data to the FCH, and then the FCH broadcasts the aggregated data to the MCH/SCH. Next, the inter-cluster transmission begins. In the three CMIMO schemes, each cluster member first transmits its data to the MCH, and the data aggregated in the MCH is then sent to the SCH, which is followed by the inter-cluster transmission. Therefore, in the four schemes, the average path hop-count for all the paths from the cluster members to the sink is obtained by *h_av_* + 2. (c) The network lifetime. After the virtual MIMO-based topology is constructed, each cluster member is allowed to send a data packet in a round (i.e., a fixed time). A FCH aggregates all the data packets from the cluster members in its cluster into one data packet, and then sends it to the MCH/SCH in its cluster. The MCH/SCH will forward it to the sink (in the manner of one-hop or multi-hop) through inter-cluster communications. In our simulation, the network lifetime is defined as the first death of MCHs in the network since the death of any MCH destroys the network topology. If a FCH or SCH first dies, the MCH in its cluster takes over its job, and thus the cluster structure has not been destroyed. When the first death of MCHs occurs, the number of sending rounds denotes the lifetime of the network. (d) The network connectivity ratio. The metric is the ratio of the number of nodes connected to virtual MIMO-based topology to the number of total nodes. (e) The average path delay for all the paths from the cluster members to the sink. The metric is computed by the formula $t_{av}=\frac{1}{\left|N\right|}\sum_{i\in N}t_{i}$, where *t_i_* is the end-to-end delay of the path from the cluster member *i* to the sink. (f) The average packet delivery ratio for all the paths from the cluster members to the sink. The metric is obtained by the formula $d_{av}=\frac{1}{\left|N\right|}\sum_{i\in N}d_{i}$, where *d_i_* is the packet delivery ratio of the path from the cluster member *i* to the sink, which is defined as the ratio of the number of delivered data packet to the destination from the cluster member *i* and the number of data packet sent by the same source to the same destination.

The wireless sensor nodes are randomly deployed in a square of length 1000 m. The sink is located to the right of the square, at a horizontal distance of 500 m and a vertical distance of 0 m from the center of the field. Without impacting all comparison objects based on the same assumptions, the parameter values used in our simulation are listed in Table 2, where we determine the ranges for these parameters based on the ranges of related simulation parameters in [1,2,3,8,27,28,29].

The simulation experiments are carried out by using OMNeT++ 4.1 network simulator [33]. In the simulation settings, each node (e.g., node *i*) employs $f\left(\gamma_{i,j}\right)={\left(1-e^{-0.5\gamma_{i,j}}\right)}^{M}$ in [34] to estimate the frame success rate in the link from it to its neighbor (e.g., node *j*), where *M* denotes the frame length, and *γ_i,j_* is the Signal to Interference Noise Ratio (SINR) of node *j*. The frame length *M* is set as 2500 bit, and the setting of value for *γ_i,j_* should ensure that the bit error rate in the link *i*→*j* is not greater than 10^−5^. Based on the frame success rate in the link *i*→*j*, the link delay (e.g., *t_i,j_*) can be estimated by ti,j=tjbf(γi,j) in [26], where $t_{j}^{b}$ denotes the packet forwarding capacity of node *j*, and is set as 100 ns/bit. The following describes and analyses the experiment results in detail.

### 4.3. Simulation Results and Analysis

In the first group of simulations, we study the impact of the number of nodes (that is, it is node density since the simulation field is fixed) on the average transmission power, the average path hop-count, the network lifetime, the network connectivity ratio, the average path delay, and the average packet delivery ratio for the ICV-MIMO and the three CMIMO schemes (denoted as CMIMO-S, CMIMO-M, and CMIMO-L). In the ICV-MIMO, *μ*, *w_e_* and *w_r_* take 0.33, 0.5 and 0.5 respectively (i.e., *μ=* 0.33, *w_e_* = 0.5 and *w_r_* = 0.5). For the three CMIMO schemes, the difference is only the value of clustering range, where the clustering ranges of CMIMO-S, CMIMO-M, and CMIMO-L take 60 m, 80 m, and 100 m respectively.

**Figure 2:**
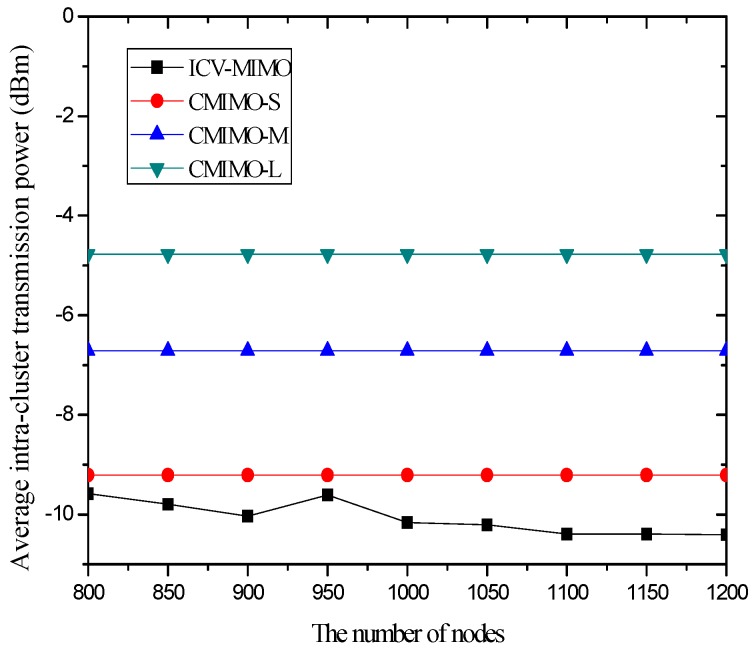
Average intra-cluster transmission power for the four schemes under the different number of nodes.

Figure 2 shows the impact of the number of nodes on the average transmission power. The results illustrate that, in the three CMIMO schemes, the average transmission power is not affected by the number of nodes. This is because all the cluster members in the same cluster employ the same transmission power corresponding to a given clustering range. In the ICV-MIMO scheme, the average transmission power decreases with the number of nodes. This is due to the fact that, when the more nodes are located in the same cluster, the average distance to the cluster head is shorter due to the more cluster members near the cluster head.

The average transmission power of ICV-MIMO is significantly lower than that of CMIMO-L. The main reason is that, on the one hand, the average clustering range of ICV-MIMO is smaller than that of CMIMO-L, on the other hand, in the ICV-MIMO scheme, each cluster member’s transmission power can be adjusted, which may be smaller than the transmission power corresponding to a given clustering range.

When compared to the CMIMO-S scheme, the advantage of ICV-MIMO is obviously reduced. This is because the average clustering range of ICV-MIMO is larger than that of CMIMO-S. The comparison between ICV-MIMO and CMIMO-M should be fairer since the size of the two scheme’s clustering ranges is very close, where the ICV-MIMO significantly outperforms the CMIMO-M in the average transmission power, and the superiority of ICV-MIMO over CMIMO-M becomes more apparent when the number of nodes is larger.

**Figure 3:**
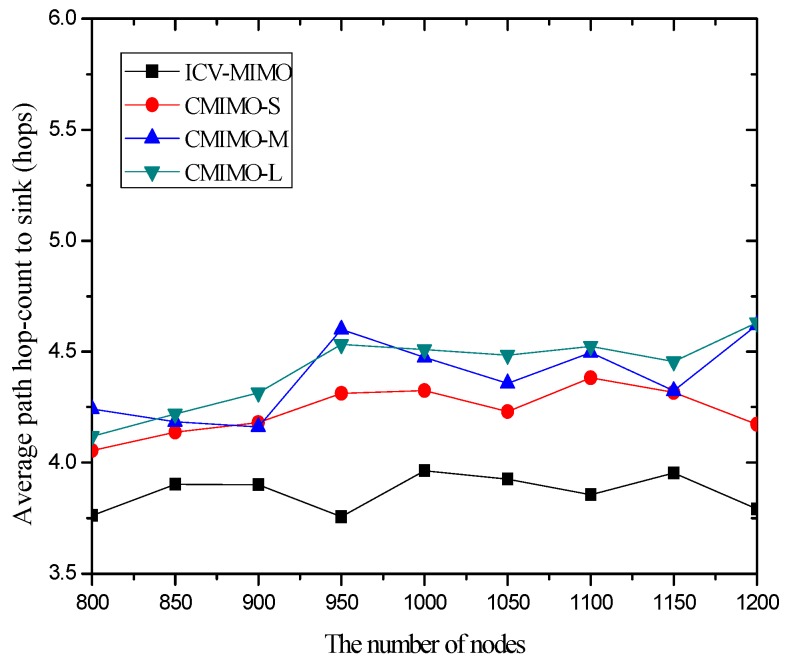
Average path hop-count to sink for the four schemes under the different number of nodes.

Figure 3 shows that the variations in the number of nodes in the same simulation area lead to slight fluctuations in the average path hop-count. This is because the previous MCH node may be replaced by the new added node with the best MCH eligibility metric, which owns larger or smaller maximum transmission range with the growing number of nodes, and thus there are the small fluctuations in the inter-cluster topology.

Also, Figure 3 reveals that a clustering range has the impact on the number of hops between the sending source CHs and the sink. One can observe that the CMIMO-L has the most path hop-count while the CMIMO-S has the smallest one among the three CMIMO schemes. The primary reason is that a smaller clustering range leads to the more number of clusters in the same network (e.g., CMIMO-S), and the number of neighboring MCHs of any MCH tends to increase under the same maximum transmission range. Therefore, a sending source MCH with more neighboring MCHs is more likely to find a shorter path to the sink.

As expected, the ICV-MIMO scheme significantly outperforms the three CMIMO schemes in the average path hop-count. The main reason is that the ICV-MIMO scheme considers nodes’ maximum transmission range to elect MCHs, and thus has the advantage of coverage over the three CMIMO schemes.

**Figure 4:**
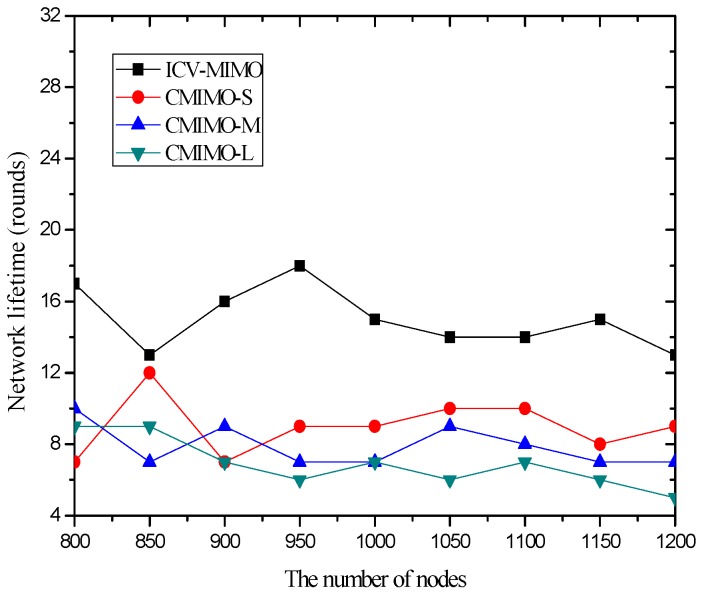
Network lifetime for the four schemes under the different number of nodes.

Figure 4 compares the network lifetime under the ICV-MIMO scheme and the three CMIMO schemes using a network lifetime definition: the time until the death of the first MCH. From Figure 4, one can observe that the ICV-MIMO scheme results in a longer network lifetime than the three CMIMO schemes. This is due to the fact that a FCH shares the majority of the data collection and data aggregation for its MCH. Therefore, the energy consumption is more balanced in the ICV-MIMO scheme than that in any of the three CMIMO schemes.

Also, Figure 4 reveals that a smaller clustering range leads to a longer network lifetime among the three CMIMO schemes. This is because there is the smaller load of data collection and data aggregation in the smaller cluster, and thus the total energy consumption for data collection and data aggregation is distributed on the more CHs, which better alleviates the overhead of individual CH. Although a cluster may forward the aggregated data for the more other clusters as the number of clusters increases, there is not any load for any FCH. However, some MCHs need to take the more loads. Usually, a MCH has an advantage over its corresponding FCH in terms of the energy since it is first elected, and also the forwarded data is reduced greatly due to aggregation, so a MCH lifetime will not be affected significantly, which is usually longer than that of its corresponding FCH.

**Figure 5:**
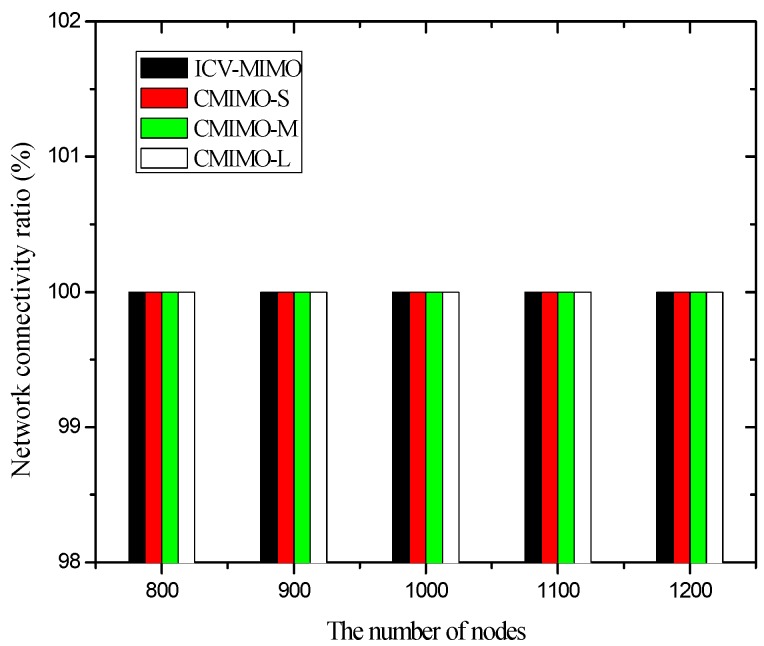
Network connectivity ratio for the four schemes under the different number of nodes.

Figure 5 shows that the four schemes can ensure network connectivity under the parameters of the first group of simulations. This is because these parameters ensure the clustering ranges of the four schemes are sufficiently small, which does not have impact on network connectivity.

For the CMIMO scheme, if the clustering range is set too large, the network connectivity ratio may be less than 100%. The main reasons are as follows. First, the larger clustering range needs the larger average inter-cluster transmission range to ensure inter-cluster connection, that is, the elected MCH communicating with neighboring MCH depends on a greater transmission ability with a larger clustering range, otherwise it disconnects. Second, the CMIMO scheme elects a MCH only according to RBLs of nodes, and thus the elected MCH may have the highest RBL value but the smallest transmission range, which may make a sending source MCH not to find any path to the sink in inter-cluster transmission period. Third, the larger clustering range leads to the higher probability that the nodes with very small transmission ranges cannot communicate with the CHs when they are located in the edge of the cluster in intra-cluster transmission period.

Since the clustering range is assigned before the execution of the CMIMO scheme, the above cases may occur due to the lack of network information (e.g., the distribution of nodes’ maximum transmission ranges). In the ICV-MIMO scheme, the above cases do not occur since the clustering ranges are determined automatically through the mutual exchange of messages.

**Figure 6:**
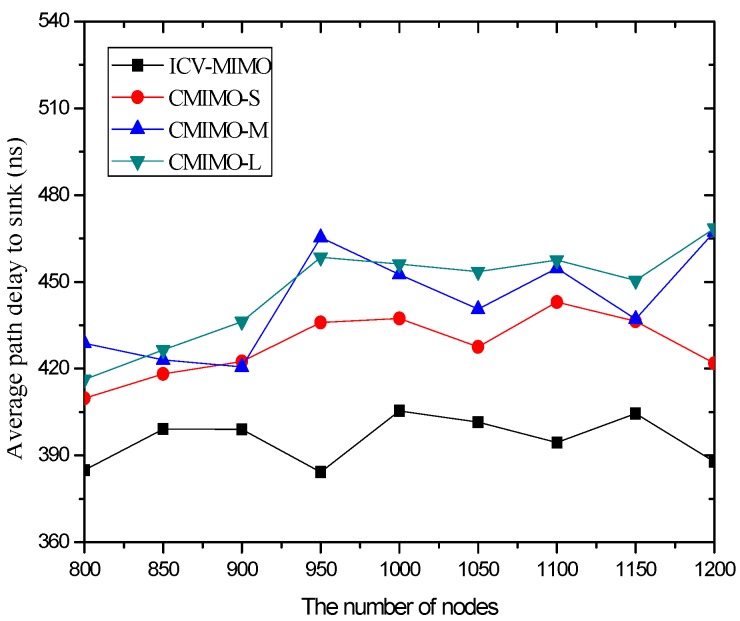
Average path delay for the four schemes under the different number of nodes.

**Figure 7:**
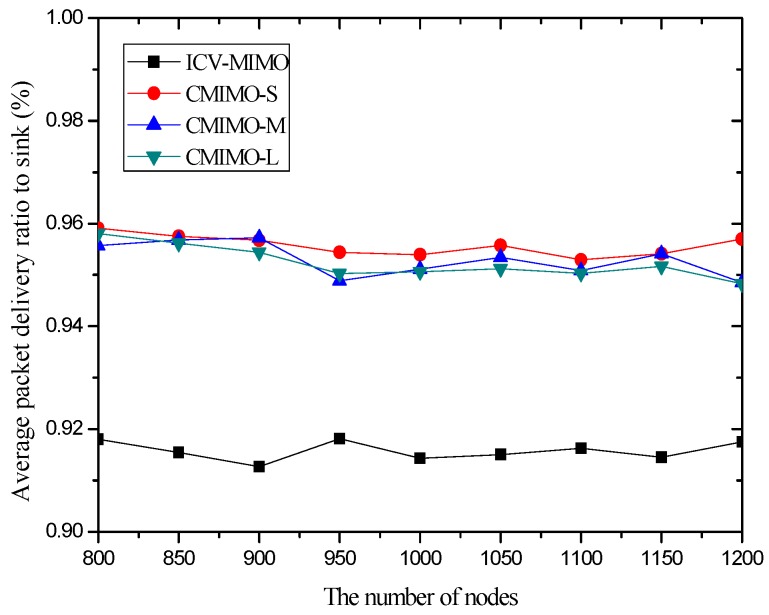
Average packet delivery ratio for the four schemes under the different number of nodes.

Figure 6 and Figure 7 illustrate that the ICV-MIMO scheme outperforms the three CMIMO schemes in terms of average path delay while it is not superior to them with respect to average packet delivery ratio. Since the three CMIMO schemes employ the same transmission power in in intra-cluster transmission period, when this transmission power ensures that the bit error rate of any link between any cluster member located at the edge of the cluster and the cluster head is not greater than a predefined value (e.g., 10^−5^), the bit error rate of any link between any cluster member near the cluster head and the cluster head will be less than this predefined value. Also, for each inter-cluster link, the bit error rate can be ensured to be the same by adjusting inter-cluster transmission power in the four schemes. Therefore, the ICV-MIMO scheme achieves a greater improvement of energy efficiency at the cost of the slight performance degradation in terms of average packet delivery ratio. Since the ICV-MIMO scheme has the slightly higher average bit error rate in intra-cluster links, it has the slightly higher average link delay in intra-cluster communications. However, as shown in Figure 3, due to the shortest average path hop-count in the four schemes, the ICV-MIMO scheme has the lowest average path delay among them.

As shown in Figure 6 and Figure 7, in the three CMIMO schemes, their average path delays have a more obvious difference than their average packet delivery ratios. This is because path delay is greatly affected by path hop-count, which is basically in line with the change of path hop-count. Due to the predefined value of low bit error rate, the difference of average bit error rate in intra-cluster links for the three CMIMO schemes is very small, and thus there is a small difference between their average packet delivery ratios in the paths from the cluster members to the sink.

**Figure 8:**
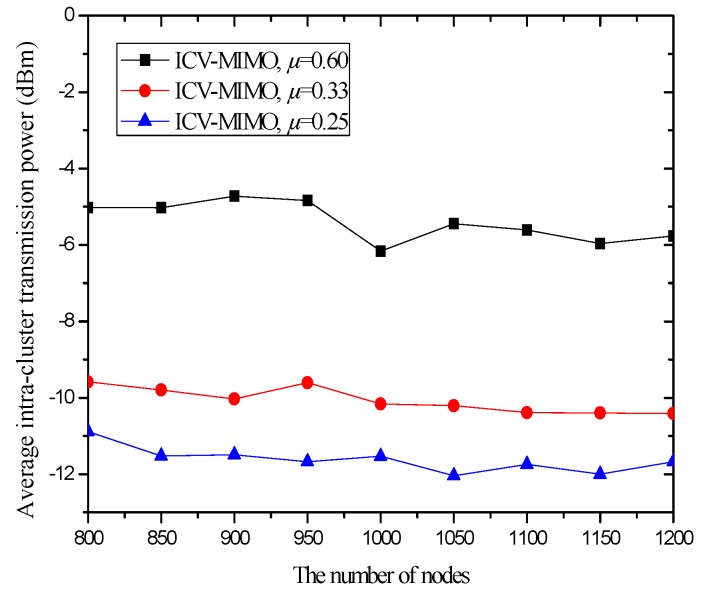
Average intra-cluster transmission power for the ICV-MIMO scheme under the different typical values for *μ*.

In the second group of simulations, we study the impact of the number of nodes on the average transmission power, the average path hop-count, the network lifetime, the network connectivity ratio, the average path delay, and the average packet delivery ratio for the ICV-MIMO scheme under the different typical values for *μ*, where *w_e_* and *w_r_* take 0.5 and 0.5 respectively. From Figure 8, one can observe that, the average transmission power increases with the value for *μ*. This is due to the fact that the average clustering range increases with the value for *μ*.

**Figure 9:**
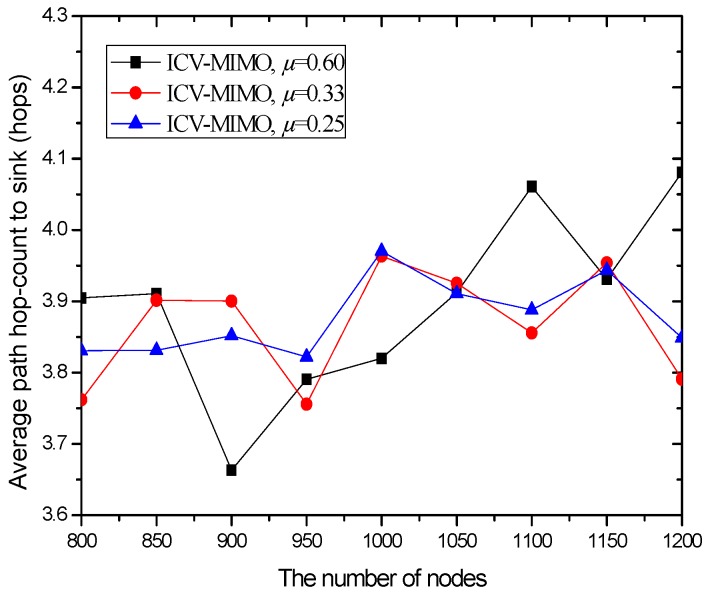
Average path hop-count to sink for the ICV-MIMO scheme under the different typical values for *μ*.

From Figure 9, one can observe that, when the value for *μ* gets smaller, the average path hop-count has smaller fluctuations with the variations in the number of nodes in the same simulation area. This may be because, on the one hand, the smaller *μ* leads to the smaller clustering range which accommodates the smaller number of nodes, and the smaller variations in the number of nodes has less impact on the replacement of MCH; on the other hand, the smaller clustering range generates more neighboring MCHs for each elected MCH node. Therefore, these factors are conducive to the stability of the path.

**Figure 10:**
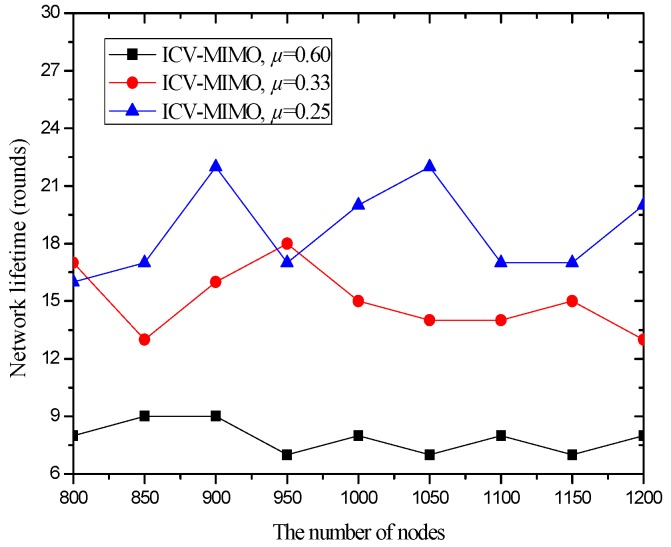
Network lifetime for the ICV-MIMO scheme under the different typical values for *μ*.

Figure 10 shows that the network lifetime decreases with the value for *μ*. The main reason is that, on the one hand, when the value for *μ* is relatively small, the average number of neighboring MCH is relatively large and thus the connectivity between the clusters is not easy to be destroyed; on the other hand, since the smaller clustering range accommodates the smaller number of nodes, the load of single cluster is smaller, where total energy consumption is shared to more clusters and thus the network energy consumption is balanced further.

**Figure 11:**
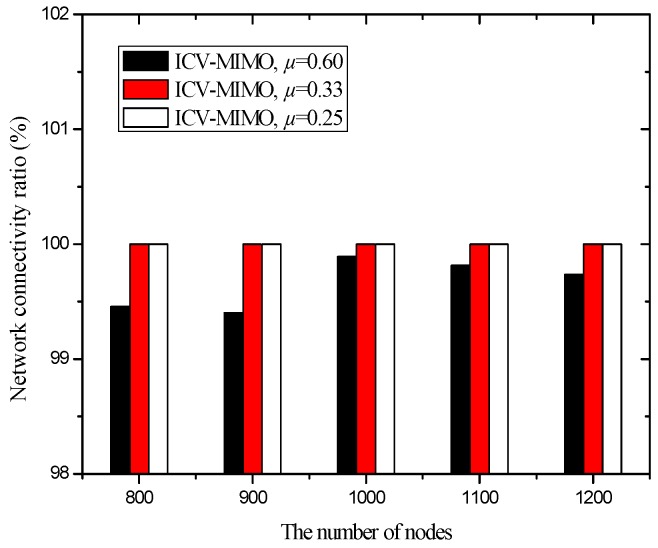
Network connectivity ratio for the ICV-MIMO scheme under the different typical values for *μ*.

Figure 11 reveals that the network connectivity ratio is less than 100% when the value for μ takes 0.6. This is because the larger μ leads to the larger clustering range. However, the network connectivity ratio is still more than 99%.

**Figure 12:**
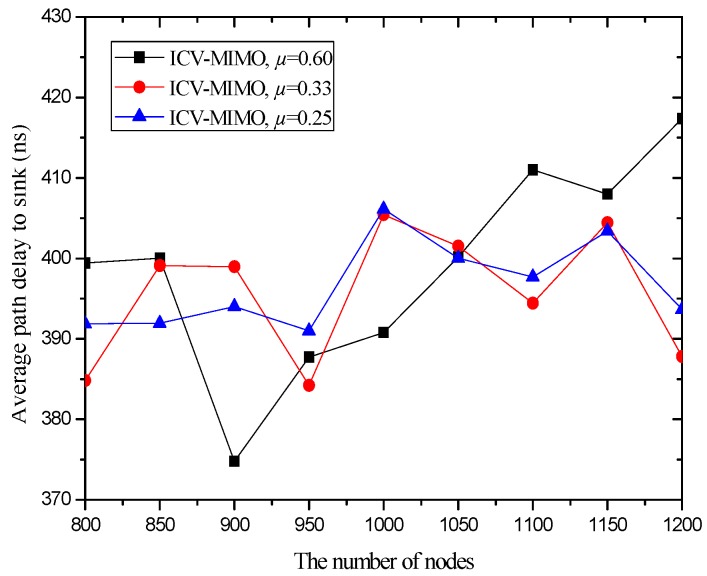
Path delay for the ICV-MIMO scheme under the different typical values for *μ*.

**Figure 13:**
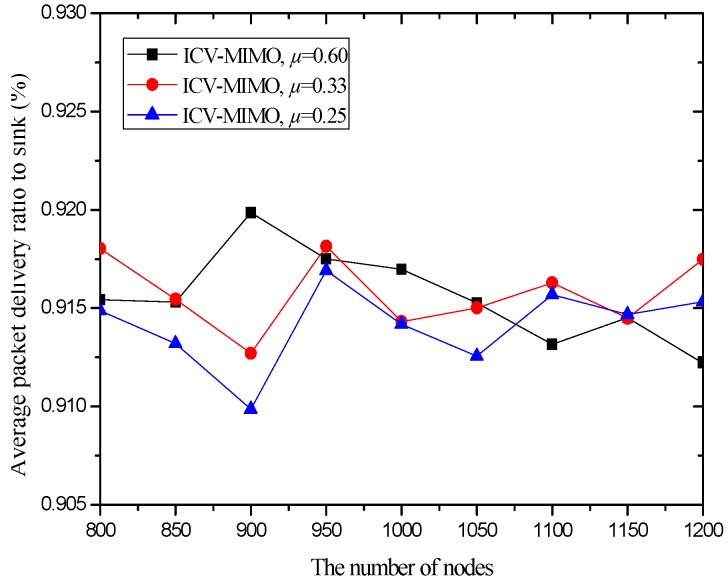
Packet delivery ratio for the ICV-MIMO scheme under the different typical values for *μ*.

The change trend of curves in Figure 12 is basically consistent with that in Figure 9, which further shows that path delay is mainly affected by path hop-count when the bit error rate of link is strictly controlled. From Figure 13, one can observe the fluctuation of curves though the absolute amount of change is very small, which may be caused by the randomness of the node distribution. Also, the curves are intertwined in Figure 13, where the main reason may be the fluctuation of path hop-count, since the bit error rate for each link is controlled to roughly the same value in inter-cluster communications.

In the third group of simulations, we study the impact of the number of nodes on the average transmission power, the average path hop-count, the network lifetime, the network connectivity ratio, the average path delay, and the average packet delivery ratio for the ICV-MIMO scheme when *w_e_* and *w_r_* take some typical values respectively, where *μ* takes 0.33.

**Figure 14:**
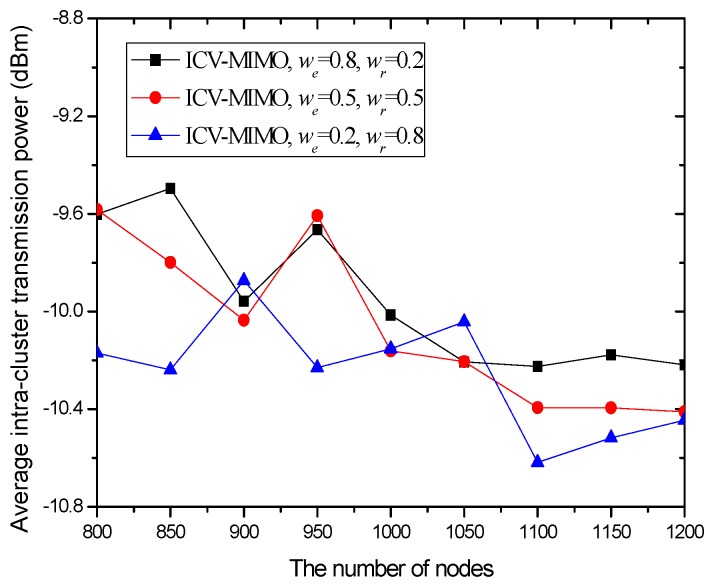
Average intra-cluster transmission power for the ICV-MIMO scheme under the different typical values for *w_e_* and *w_r_*.

The results from Figure 14 illustrates that the average intra-cluster transmission power slightly decreases with the value of *w_r_*. This is because the larger *w_r_* tends to elect a MCH with the stronger communication coverage capability, where the average clustering range computed by this MCH according to the Equations (5) and (6) tends to be smaller in general due to the participation of more nodes.

**Figure 15:**
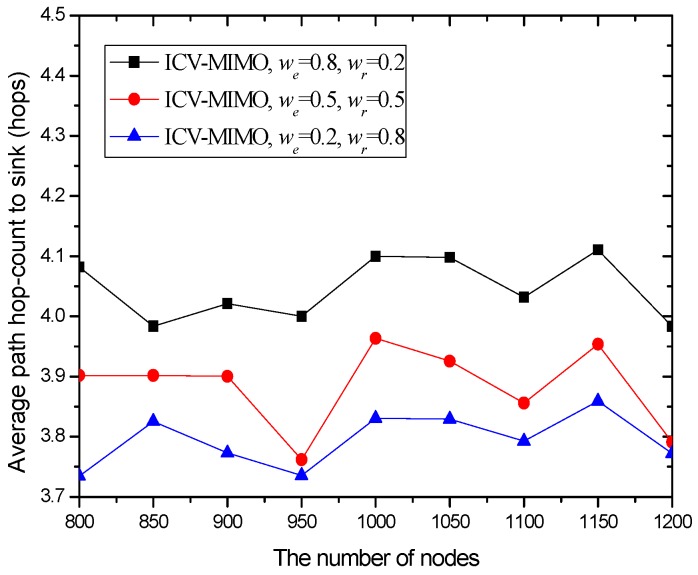
Average path hop-count to sink for the ICV-MIMO scheme under the different typical values for *w_e_* and *w_r_*.

That is, for the average intra-cluster transmission power shown in Figure 14, its maximum value is limited by the clustering range. When *w_r_* gets larger, the elected MCH’s approximate maximum transmission range is larger, and thus more nodes are in this coverage. All these nodes’ approximate maximum transmission ranges are used to compute an average value by using the Equation (5), which tends to lead to a smaller average value that is clustering range when the number of these nodes is larger. Therefore, one observes a decreasing trend with *w_r_* in Figure 14. Also, the smaller clustering range generates the more clusters and thus there are the more neighboring MCHs for each MCH with the stronger communication coverage capability. This makes a source MCH has the higher possibility to find the shorter path to the sink, which is confirmed by the results shown in Figure 15.

In other words, for the average path hop-count to sink shown in Figure 15, this value is affected by the density of network graph consisting of MCHs. As mentioned above, when *w_r_* gets larger, the average approximate maximum transmission range tends to reduce, and thus clustering range gets smaller and the number of clusters increases. Also, each MCH has the higher number of neighboring MCHs if it has a larger approximate maximum transmission range. Therefore, the network graph consisting of MCHs is denser, and it is more likely to find a shorter hop-count path to the sink. So, one observes a decreasing trend with *w_r_* in Figure 15.

**Figure 16:**
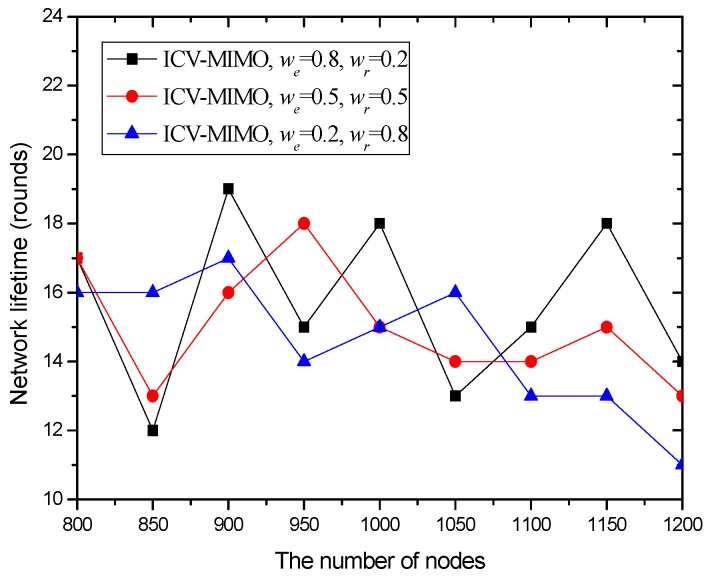
Network lifetime for the ICV-MIMO scheme under the different typical values for *w_e_* and *w_r_*.

Figure 16 shows that the network lifetime slightly increases with the value of *w_e_*. The main reasons are that the larger value for *w_e_* leads to the MCHs with the higher energy and thus the connectivity between the clusters is kept longer. Also, when the value for *w_e_* is relatively smaller, the relatively larger value for *w_r_* suppresses the deterioration of the network lifetime, so one can observe that the difference between the three curves in Figure 16 is not obvious.

In a word, for the network lifetime shown in Figure 16, this value is mainly related to residual energy level of MCHs. When *w_e_* gets larger, the elected MCHs have higher energy level and thus the network lifetime is longer. On the other hand, when *w_e_* gets smaller, although the elected MCHs have relatively lower energy level, the network lifetime will not be significantly worse. This is because as *w_e_* gets smaller, *w_r_* gets larger accordingly, which leads to the lower average intra-cluster transmission power (as shown in Figure 14). Therefore, to a certain extent, it is able to compensate for the impact of the lower remaining energy on the network lifetime.

**Figure 17:**
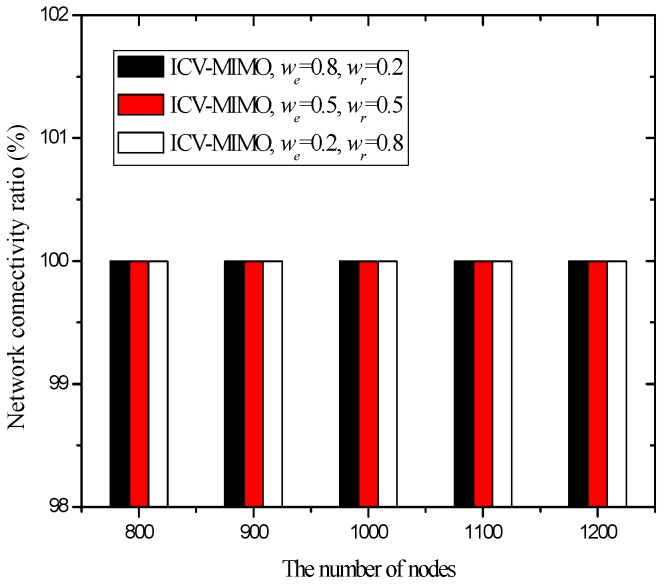
Network connectivity ratio for the ICV-MIMO scheme under the different typical values for *w_e_* and *w_r_*.

Figure 17 shows that the network connectivity ratio is hardly affected by the value for w_e_ or w_r_. As shown in Figure 11, the network connectivity ratio is more than 99% but less than 100% under μ’s taking 0.6. Therefore, it is sufficient for μ’s taking 0.33 to ensure network connectivity.

**Figure 18:**
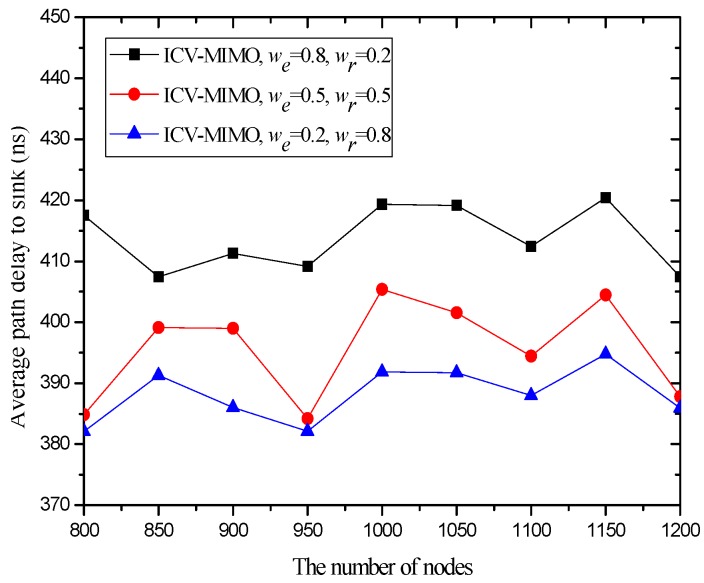
Path delay for the ICV-MIMO scheme under the different typical values for *w_e_* and *w_r_*.

**Figure 19:**
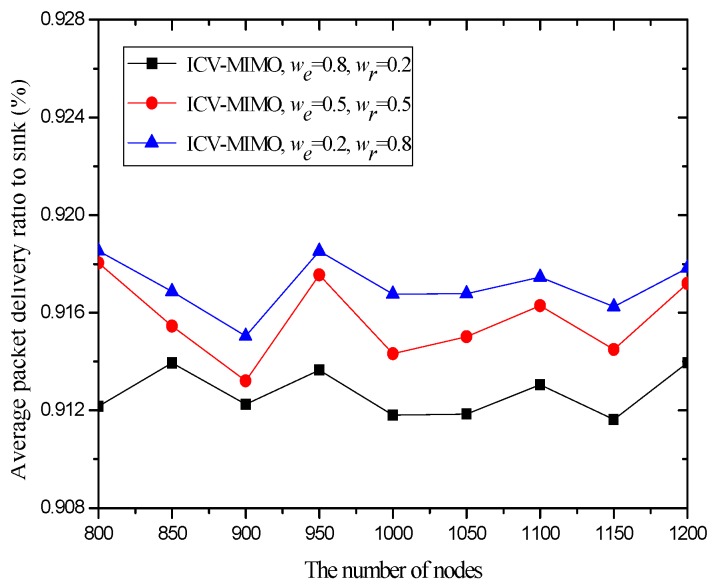
Packet delivery ratio for the ICV-MIMO scheme under the different typical values for *w_e_* and *w_r_*.

Figure 18 reveals the similar change trend of curves to that in Figure 15, which has the same reasons as those explained in the related part of the second group of simulations. A path with a smaller hop-count usually has a higher packet delivery ratio, since the frame success rate for each link of path is at most 100% and usually less than 100%. As shown in Figure 15, the ICV-MIMO scheme with *w_e_* = 0.2 and *w_r_* = 0.8 has the shortest average path hop-count, while the ICV-MIMO scheme with *w_e_* = 0.8 and *w_r_* = 0.2 has the longest one. Therefore, from Figure 19, one can see that the former has the highest packet delivery ratio while the latter has the lowest one.

In Figure 2, Figure 3, Figure 4, Figure 5, Figure 6, Figure 7, Figure 8, Figure 9, Figure 10, Figure 11, Figure 12, Figure 13, Figure 14, Figure 15, Figure 16, Figure 17, Figure 18 and Figure 19, each data point in each plot represents an average of 10 simulation runs, and the simulation process continues until the first death of MCHs occurs. In addition, although there is not randomness (e.g., node movement and channel fading) in our simulation, there is still randomness in terms of the node density distribution and the maximum transmission power of nodes. Therefore, the plots of part simulation results go up and go down in a certain number of nodes.

In addition, although there is only a factor (i.e., the number of nodes) on the *X* axis in our simulation diagrams, we implicitly consider the other factors (e.g., node density, inter-cluster range, traffic intensity), as mentioned in [3]. The simulation field is fixed in our simulation settings, so the change of the number of nodes reflects the change of node density.

The simulation results of the ICV-MIMO scheme under the different values of the parameter *μ* also reflect the simulation performance of the different inter-cluster ranges. When the parameter *μ* gets large, the required distance for inter-cluster connectivity is also large, and the six metrics also have some changes accordingly. Since each cluster member must send a packet to the sink, traffic intensity increases with the number of nodes. Therefore, the factor “traffic intensity” is considered implicitly in our simulations.

## 5. Conclusions

We proposed an improved clustering scheme for Virtual MIMO-based topology construction (ICV-MIMO) to determine adaptively both the inter-cluster transmission modes and the clustering ranges. The ICV-MIMO scheme partitions a WSN into a number of clusters that have at most three CHs per cluster. The cluster head selection criterions consider both nodes’ RBLs and nodes’ approximate maximum transmission capability. Each cluster member’s transmission power is optimized according to the distance between it and CHs. The added FCHs amortize the cooperation overhead of MCHs while they do not involve more clustering overhead. Also, the problem of unreasonable clustering range setting can be avoided. The simulation results indicate that the ICV-MIMO scheme achieves significant reduction in energy consumption and thus has longer network lifetime, compared with the existing typical virtual MIMO-based scheme.

Our work considered only SISO mode for the cluster members regardless of the distances from them to CHs. In order to further improve the energy efficiency, a possible future extension is to incorporate SIMO mode in intra-cluster communications, i.e., allows the cluster members to dynamically switch their transmission modes between SISO and SIMO, depending on energy consumption consideration. In addition, in order to stimulate the cooperation of individual nodes, introducing the game-theoretic-based incentive methods [35,36] into our scheme is also an interesting future extension. Also, the research of event detection methods (e.g., failure detection [37], bursty workload detection [38], target detection [39], attack detection [40]) for distributed systems has been a concern of researchers, which can guarantee continuous, safe, secure, and dependable operation in distributed systems. Thus, in order to achieve the goal for continuous, safe, secure, and dependable operation, it is worthwhile to consider an event detection mechanism for our scheme in future works.

## Figures and Tables

**Table 1:** The overhead comparison between the clustering schemes.

Scheme	Message Overhead	Time Overhead
ICV-MIMO	*O(N_nei_)*	*max*{*O*(*T_t_*)*, O*(*N_nei_·T_r_*)}
CMIMO	*O*(*N_deg_*)	*max*{*O*(*T_t_*)*, O*(*N_deg_·T_r_*)}

**Table 2:** The simulation parameters.

Description	Parameter	Value
Transmitting antenna gain	*G_t_*	1
Receiving antenna gain	*G_r_*	1
Transmitting antenna height	*h_t_*	1 m
Receiving antenna height	*h_r_*	1 m
Maximum transmitting power	*p_i,max_*	For any node *i*, *p_i,max_* is set as randomly distributed between 0 dBm and 20 dBm
Receiver sensitivity	*p^h^_i_*	For any node *i*, *p^h^_i_* is as −85 dBm
Carrier signal wavelength	λ	0.1224 m
System loss factor	*L*	1
Crossover distance	*d_crossover_*	103 m
Initial battery capacity	*e_i,int_*	For any node *i*, *e_i,int_* is randomly distributed between 0.05 J and 0.2 J
Bit rate	*R_b_*	2 Mbit/s
SNR threshold	*γ*(*M_t_*,*M_r_*)	*γ*(1,1) = 54.4 dBm; *γ*(1,2) = 40.6 dBm; *γ*(2,1) = 44.1 dBm; *γ*(2,2) = 36.9 dBm
Factor depending on amplifier drain efficiency and underlying modulation	*δ*	0.5
Single-sided thermal noise PSD	*N_o_*	−171 dBm/Hz
Passband bandwidth	*B*	10 kHz
Receiver noise figure	*N_f_*	40 dBm
Constant depending on transmitter and receiver antenna gains	*G_o_*	1
Link margin compensating for hardware variations and other sources of interference	*M_l_*	40 dBm
Power consumption for digital-to-analog converter	*P_DAC_*	12 dBm
Power consumption for mixer	*P_mix_*	15 dBm
Power consumption for low noise amplifier	*P_LNA_*	13 dBm
Power consumption for intermediate frequency amplifier	*P_IFA_*	3 dBm
Power consumption for active filter at the transmitter side	*P_filt_*	4 dBm
Power consumption for active filter at receiver side	*P_filr_*	4 dBm
Power consumption for analog-to-digital converter	*P_ADC_*	12 dBm
Power consumption for frequency synthesizer	*P_syn_*	17 dBm
Length of data packet consisting of 4 sub-packages	*l*	10,000 bit
Reference energy	*e_ref_*	0.2 J
Reference distance	*d_ref_*	420 m
